# Fetal glucose availability: a key regulator of the metabolic, hormonal and contractility profiles of the fetal sheep heart

**DOI:** 10.1113/JP288303

**Published:** 2025-05-28

**Authors:** Melanie R. Bertossa, Jack R. T. Darby, Stacey L. Holman, Steven K. S. Cho, Ashley S. Meakin, Mitchell C. Lock, Jordan A. Minns, Michael D. Wiese, Christopher K. Macgowan, Mike Seed, Janna L. Morrison

**Affiliations:** ^1^ Early Origins of Adult Health Research Group, Health and Biomedical Innovation, UniSA: Clinical and Health Sciences University of South Australia Adelaide South Australia Australia; ^2^ Department of Physiology, Faculty of Medicine University of Toronto Toronto Canada; ^3^ Translational Medicine Hospital for Sick Children Toronto Canada; ^4^ Department of Medical Biophysics, Faculty of Medicine University of Toronto Toronto Canada; ^5^ Division of Cardiology Hospital for Sick Children Toronto Canada; ^6^ Department of Paediatrics, Faculty of Medicine University of Toronto Toronto Canada

**Keywords:** cardiovascular disease, fetal growth restriction, fetus, late gestation, left ventricle, maternal undernutrition

## Abstract

**Abstract:**

There is an association between fetal growth restriction (FGR) and a poor lifetime cardiac health trajectory. Defining the underlying mechanisms will aid in developing interventions to decrease the contribution of FGR‐born offspring to the global burden of cardiovascular disease. One cause of FGR is maternal undernutrition. In late‐gestation undernutrition (LGUN) fetal glucose supply, a main energy source for the fetal heart, is reduced. This may be a key contributor to altered fetal cardiac development; thus restoration of fetal glucose availability in the LGUN setting may be a viable target for intervention. To investigate the role of glucose availability in fetal heart development, we utilized an established pregnant sheep model of LGUN (50% global nutrient restriction) with or without a continuous intrafetal glucose infusion. LGUN reduced fetal plasma glucose concentrations, resulting in brain sparing that was normalized by intrafetal glucose infusion. LGUN decreased the protein abundance of oxidative phosphorylation complexes 1 and 3; however glucose infusion returned complex 3 abundance to that of controls. LGUN increased the phosphorylation of contractility and hypertrophy marker CAMKII, which was associated with increased left ventricular cardiac output; however intrafetal glucose infusion normalized CAMKII. Our findings demonstrate that glucose plays a specific role in regulating cardiac development *in utero*, highlighting the importance of adequate maternal nutrition in late gestation.

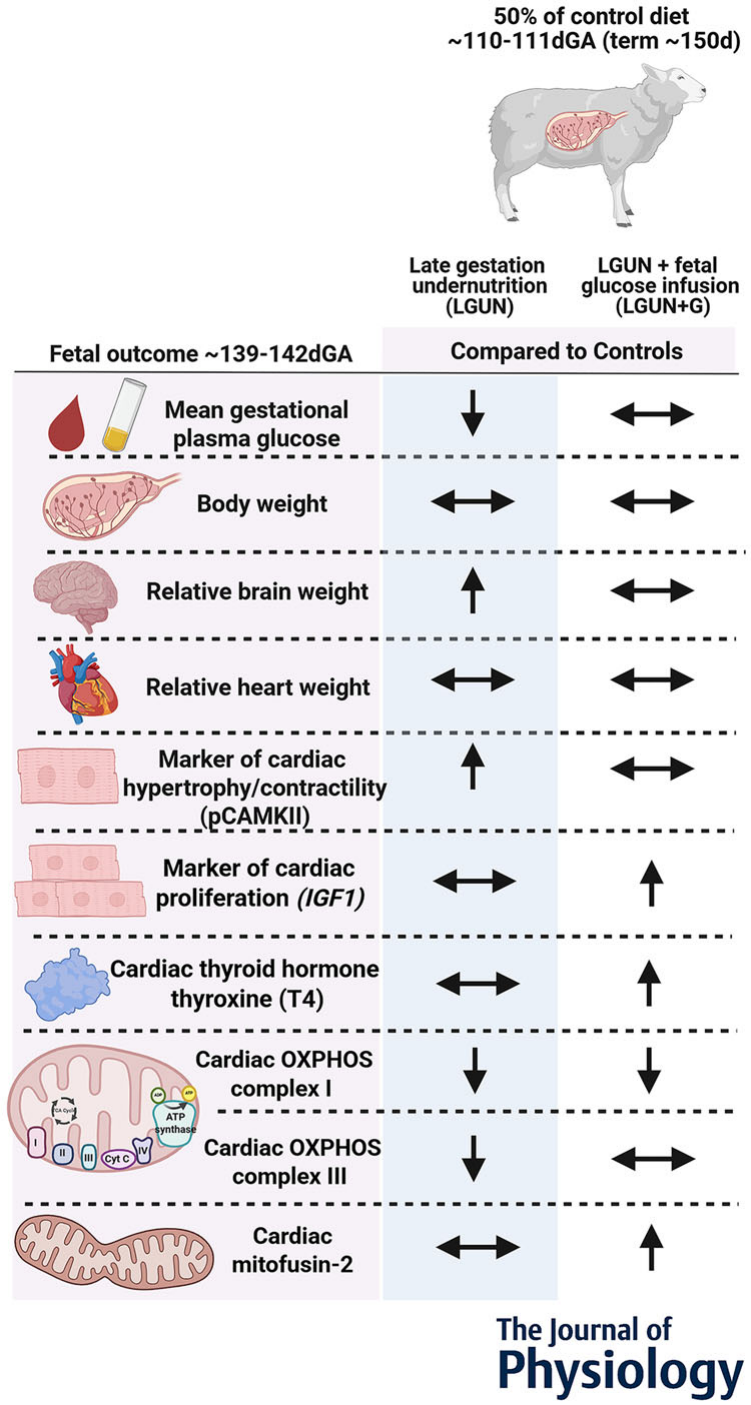

**Key points:**

- Maternal late‐gestation undernutrition (LGUN) reduces fetal plasma glucose concentrations. To investigate the role of glucose availability in fetal left ventricle (LV) development, we assessed whether LGUN‐induced alterations in the contractility, metabolic and hormonal profiles can be ameliorated in LGUN fetuses receiving glucose infusion (LGUN+G).
- Relative brain weight was increased in LGUN compared to controls and restored in LGUN+G despite fetal glucose infusion only partially normalizing fetal plasma glucose concentrations to that of controls.
- LGUN decreased cardiac oxidative phosphorylation (OXPHOS)complex 1 and 3 abundance, and LGUN+G restored complex 3 to that of controls.
- LGUN increased the activation of the contractility marker, Ca^2+^/calmodulin‐dependent protein kinase II (p‐CAMKII), but restored in LGUN+G. The magnetic resonance imaging measure of the LV cardiac output was positively correlated with p‐CAMKII expression in LGUN.
- This study highlights the role of *in utero* glucose availability in regulating the abundance of fetal LV OXPHOS complex 3 and CAMKII activation *in utero*.

## Graphical abstract

**Abstract figure legend** To investigate the role of glucose availability in fetal left ventricle (LV) development, this study assessed whether maternal late gestation undernutrition (LGUN; 50% of Control diet) induced alterations in the contractility, metabolic, and hormonal profile can be ameliorated in LGUN fetuses receiving glucose infusion (LGUN+G). Relative brain weight was increased in LGUN compared to Control and restored in LGUN+G, despite fetal glucose infusion only partially normalising fetal plasma glucose concentrations to that of Controls. LGUN and LGUN+G had no impact on relative heart weights. LGUN increased the activation of the contractility marker, Ca2+/calmodulin‐ dependent protein kinase II (p‐CAMKII), but restored in LGUN+G. LGUN had no impact on cardiac concentrations of thyroid hormone thyroxine (T4) and the gene expression of insulin‐ like growth factor 1 (IGF1); however, both were increased in LGUN+G. LGUN decreased cardiac oxidative phosphorylation (OXPHOS) complex 1 and 3 abundance, and LGUN+G restored complex 3 to that of Controls. LGUN had no impact on mitofusin‐ 2, a marker of mitochondrial fusion; however, it was increased in LGUN+G. This study highlights the role of *in utero* glucose availability in regulating key aspects of the fetal contractility, metabolic, and hormonal profiles.

## Introduction

Fetal growth restriction (FGR, birth weight <10th centile for gestational age) is a significant risk factor for the development of hypertension, ischaemic heart disease and associated co‐morbidities in later life (Barker, 2000; Barker et al., 1989). FGR is often caused by placental insufficiency and associated with inadequate placental perfusion, leading to reduced transfer of oxygen and nutrients from maternal to the fetal circulation, with fetal hypoglycaemia and hypoxaemia being hallmarks of FGR (Economides & Nicolaides, 1989; Economides et al., 1991; Owens, 1995; Robinson et al., 1994; Soothill et al., 1987). Although preclinical models of placental insufficiency have provided insights into the impact of hypoglycaemia and hypoxaemia on fetal cardiac development, the independent role of hypoglycaemia in the absence of hypoxaemia remains unclear. FGR can also result from maternal nutrient restriction (MNR) despite a normoxaemic *in utero* environment, and understanding how hypoglycaemia without hypoxaemia contributes to the fetal origins of cardiovascular disease (CVD) is important, given the global prevalence of MNR due to food poverty or eating disorders during pregnancy (das Neves et al., 2022; Easter et al., 2013; Santos et al., 2017).

In preclinical models of placental insufficiency‐induced FGR, maturation of cardiomyocytes (CMs, the functional units of the heart) is delayed with fewer but larger CMs in late gestation, an observation that remains evident a year after birth in lambs born with a low birth weight (Botting et al., 2014; Bubb et al., 2007; Louey et al., 2007; Morrison et al., 2007; Vranas et al., 2017; Wang et al., 2011). Reduced CM endowment may be due to increased apoptosis of CMs that are not replaced, which coincides with reduced expression of markers of CM proliferation (e.g. reduced circulating insulin‐like growth factor (IGF) 1 and cardiac IGF signalling) (Eremia et al., 2007; Owens et al., 1994; Zhang et al., 2024). The independent contributions of hypoxaemia or hypoglycaemia, as well as their potentially synergistic effects on reduced CM endowment in FGR caused by placental insufficiency, are not fully elucidated. However in the absence of hypoxaemia, MNR‐induced hypoglycaemia reduces fetal plasma IGF1 concentrations in sheep, rodents and guinea‐pigs (Bassett et al., 1990; Dong & Thompson, 2006; Dwyer & Stickland, 1992; Gallaher et al., 1998; Jones et al., 1990; Masoumy et al., 2018; Woodall et al., 1996). MNR also increases apoptosis and reduces CM numbers (Masoumy et al., 2018), suggesting an independent role for hypoglycaemia in altered cardiac development in the FGR heart.

The well‐characterized prenatal surge in thyroid hormone (TH) in late gestation that occurs in humans and sheep is critical in regulating the appropriate timing of CM maturation (Chattergoon et al., 2023; Dimasi, Darby, et al., 2023; Forhead & Fowden, 2014; Morrison, 2008; Nathanielsz & Fisher, 1979). Thus underlying this delayed CM maturation may be reduced fetal plasma concentrations of THs in FGR (Dwyer & Stickland, 1992; Johnsen et al., 2013; Jones et al., 1990; Lingas et al., 1999; Steinhauser et al., 2021; Vonnahme et al., 2003). This may be directly related to fetal hypoglycaemia in FGR altering the fetal thyroid gland structure and the peripheral utilization of THs in late gestation (Andrianakis et al., 1990). Although hypoxaemia and hypoglycaemia in placental insufficiency‐induced FGR have no impact on cardiac concentrations of THs in late gestation (Dimasi, Darby, Cho, et al., 2023), it remains unknown whether hypoglycaemia in isolation due to MNR impacts TH concentrations within the fetal heart tissue itself. Moreover THs facilitate the normal developmental transition from utilizing glucose and lactate to fatty acids for metabolic fuel via oxidative phosphorylation (OXPHOS) in late gestation (Drake et al., 2023). Whether this is associated with their role in regulating the cardiac growth profile or vice versa is unknown (Chattergoon et al., 2023; Dimasi, Darby, et al., 2023). However it is clear that fetal hypoglycaemia and hypoxaemia reduce the abundance of OXPHOS complexes (Dimasi, Darby, Cho, et al., 2023), oxidation–reduction (REDOX) ratios (Dimasi et al., 2021) and overall mitochondrial respiration (Chang et al., 2024), and suppresses genes involved in fatty acid metabolism (Drake et al., 2022) in the fetal sheep heart in placental insufficiency. Importantly these cardiometabolic alterations are not restricted to sheep models where both oxygen and nutrients are restricted, with non‐human primate (NHP) fetal hearts exposed to MNR‐induced hypoglycaemia without hypoxaemia also exhibiting this trait (Pereira Susana et al., 2021).

To better understand the role of fetal glucose availability without the confounding effects of fetal hypoxaemia, we developed a pregnant sheep model of late‐gestation undernutrition (LGUN, 50% reduction in global maternal nutrient supply). In this model mean gestational fetal plasma glucose concentrations are reduced (Edwards & McMillen, 2001) and markers of contractility and hypertrophy (i.e. p‐CAMKII, IGF2R) are increased, alongside elevated collagen deposition in the fetal right ventricle (RV; Darby et al., 2018). The RV is functionally significant during fetal life (being responsible for ∼66% combined ventricular output), but at birth, the fetal left ventricle (LV) becomes dominant, working against higher afterload of the systemic circulation than the placenta (Rudolph, 1985). Here we hypothesized that LGUN would result in similar molecular changes in the fetal LV as previously found in the RV and that restoring fetal glucose concentrations may mitigate the impact of LGUN. Using a sheep model of LGUN, we investigated whether LGUN‐induced alterations in the contractility, metabolic and hormonal profiles of the fetal LV can be ameliorated by a continuous intrafetal glucose infusion.

## Methods

All experimental protocols were approved by the South Australian Health and Medical Research Institute (SAHMRI) Animal Ethics Committees (SAM351) and followed the Australian Code of Practice for the Care and Use of Animals for Scientific Purposes by the National Health and Medical Research Council. The experiment was designed in line with the ARRIVE guidelines (Kilkenny et al., 2010). All investigators were aware of the ethical principles that have been outlined by Grundy (2014) and the principles of the 3Rs (Russell & Burch, 1959).

### Animals and surgery

Fifty‐six pregnant Merino ewes supplied by SAHMRI Farm (Burra, Adelaide, South Australia, Australia) were housed in individual pens in animal holding rooms at a constant ambient temperature of 20–22°C and a 12‐h light–dark cycle. At ∼109–124 days’ gestation (term = 150 days), pregnant ewes underwent surgery under sterile conditions as previously described (Dyer et al., 2009; Morrison et al., 2005; Ren et al., 2021). Pregnant ewes were fasted and administered analgesia (meloxicam 0.5 mg kg^−1^, s.c., Troy Laboratories Pty Ltd,Glendenning, New South Wales, Australia) 1 day before and on the day of surgery (Varcoe et al., 2019). General anaesthesia was induced by intravenous injection of diazepam (0.3 mg kg−1, Ceva Animal Health Pty Ltd,Glenorie, New South Wales, Australia) and ketamine (5 mg kg−1 Ceva Animal Health Pty Ltd, Glenorie, New South Wales, Australia), which was maintained during surgery with 1.5%–2.5% isoflurane (Lyppards, Adelaide, South Australia, Australia) in 100% oxygen. Vascular catheters were implanted into a fetal femoral artery, femoral vein and maternal jugular vein, as well as the amniotic cavity as previously described (Dyer et al., 2009; Morrison et al., 2005; Ren et al., 2021). To allow for daily flushing of catheters postsurgery, the maternal and fetal catheters were externalized via an incision in the ewe's side. During surgery the fetus received subcutaneous injections of antibiotics: 1 ml of Duplocillin (procaine penicillin 150 mg ml^−1^, benzathine penicillin 125 mg ml^−1^, Intervet, Bendigo, Victoria, Australia) and 1 ml of dihydrostreptomycin (125 mg ml^−1^, Sigma, St. Louis, MO, USA) in sterile saline (Lyppards). Pregnant ewes received an intramuscular injection of antibiotics (3.5 ml of Duplocillin and 2 ml of 125 mg ml^−1^ dihydrostreptomycin (Sigma)) on the day of surgery and for 3 days after surgery. Fetuses received sodium ampicillin (500 mg, Alphapharm Pty Ltd, Queensland, Australia) through the amniotic catheter for 4 days postsurgery as previously described (Dyer et al., 2009; Morrison et al., 2005; Ren et al., 2021).

### Nutritional regime

At 110–111 days gestational age (dGA), ewes were randomly assigned by minimization based on maternal weight, maternal fasting blood glucose and then fetal sex to one of three groups: (i) control (*n* = 14 (8 male (m), 6 female (f))), (ii) LGUN (*n* = 13 (6m, 7f)) or (iii) LGUN + intrafetal glucose infusion (LGUN+G, *n* = 6 (5m, 1f)), and maintained on the diet until postmortem (PM, 29 ± 1 days) (Cho et al., 2024; Meakin et al., 2022; Ren et al., 2021). The diet consisted of Lucerne Chaff (15 g kg^−1^) and Ewe & Lamb Nuts (4.5 g kg^−1^), containing cereal hay, lucerne hay, barley, oats, almond shells, lupins, oat bran, lime and molasses (Johnsons & Sons, Kapunda, South Australia, Australia) with a total metabolizable energy intake for the control group of 0.17 ± 0.01 MJ kg^−1^ day^−1^, which is defined as 100% nutritional requirement (Lie et al., 2014; Lock et al., 2017; Nicholas et al., 2013). Lucerne Chaff provided 80% of the total energy requirements (8.3 MJ kg^−1^ metabolizable energy, 193 g kg^−1^ of crude protein and 85% dry matter), and 20% of the energy requirements were obtained from Ewe & Lamb Nuts (8.0 MJ kg^−1^ metabolizable energy, 110 g kg^−1^ of crude protein and 90% dry matter). The LGUN group received ∼50% reduction in the control diet (0.085 ± 0.01 MJ kg^−1^ day^−1^ as previously described; Cho et al., 2024; Darby et al., 2018; Edwards & McMillen, 2001; Meakin et al., 2022; Ren et al., 2021). The LGUN+G group received the same amount of metabolizable energy intake as the LGUN group, with constant glucose infusion through the fetal femoral vein catheter (0.57 ± 0.0086 mmol h^−1^) as previously described (Cho et al., 2024; Meakin et al., 2022; Ren et al., 2021). The feed allowance of all ewes was proportionately increased by 15% every 10 days over the experimental period to meet the increasing substrate demands of the growing fetus (Cho et al., 2024; Darby et al., 2018; Edwards & McMillen, 2001; Meakin et al., 2022; Ren et al., 2021).

### Maternal and fetal blood collection

Fetal blood (∼0.5 ml) was collected from the fetal femoral artery daily to monitor fetal health by measuring blood acidity/basicity (pH), partial pressure of oxygen (PaO_2_), partial pressure of carbon dioxide (PaCO_2_)_,_ haemoglobin (Hb), haematocrit (Hct), oxygen saturation (SO_2_) and lactate, with temperature corrected to 39°C (RAPIDPoint 500, Siemens Healthineers, Erlangen, Germany). Fetal plasma glucose concentrations were measured by enzymatic analysis using hexokinase and glucose‐6‐phosphate dehydrogenase to measure the formation of NADH photometrically at 340 nm (Konelab 20XTi, program version 6.0 automated analysis system, Thermo Fisher Scientific, Waltham, Massachusetts, USA) as previously described (Morrison et al., 2007; Muhlhausler et al., 2009). A glucose measuring system determined maternal plasma glucose (jugular vein) concentrations daily (Biosen C line, EKF Diagnostics, Cardiff, UK) (Cho et al., 2024). These daily values obtained throughout the experimental protocol were used to calculate the mean gestational maternal and fetal glucose and blood gas values.

### Measurement of fetal basal blood pressure

In a subset of fetuses basal blood pressure was recorded at 118dGA (control, *n* = 8 (5m, 3f); LGUN, *n* = 7 (3m, 4f); LGUN+G, *n =* 6 (5m, 1f)) and 138dGA (control, *n* = 8 (4m, 4f); LGUN, *n =* 6 (2m, 4f); LGUN+G, *n =* 5 (5m, 0f)) by connecting the fetal femoral artery and amniotic catheters to displacement transducers, a quad‐bridge amplifier and a data acquisition unit (PowerLab, ADInstruments, Castle Hill, New South Wales, Australia) as previously described (Danielson et al., 2005; Dyer et al., 2009). Fetal blood pressure was then corrected for amniotic pressure. Data were sampled at 1000 Hz and recorded using LabChart 7 (ADInstruments). Basal blood pressure was recorded over a 1–3 h period. Data for systolic blood pressure (SBP), diastolic blood pressure (DBP), mean arterial pressure (MAP) and heart rate (HR) were extracted in 1‐min epochs and then averaged throughout their basal recording.

### Fetal cardiac magnetic resonance imaging

In a subset of animals (control, *n* = 8 (3m, 5f); LGUN, *n =* 7 (5m, 2f); LGUN+G, *n =* 5 (5m, 0f)), fetal cardiac MRI (CMR) was performed on the day before PM (139–140dGA) to assess left ventricular function. Ewes underwent magnetic resonance imaging (MRI) scanning in a 3.0 Tesla (T) scanner (Skyra, Siemens Healthineers, Erlangen, Germany). Ewes were fasted for a minimum of 12 h before the scan and placed on their left lateral side for the scan. Fetal arterial blood samples were collected and recorded during MRI to monitor fetal health. Ewes were intubated, and general anaesthesia was induced with diazepam (0.3 mg kg^−1^, Ceva Animal Health Pty Ltd,Glenorie, New South Wales, Australia) and ketamine (5 mg kg^−1^, Ceva Animal Health Pty Ltd, Glenorie, New South Wales, Australia) and maintained with 2.0%–3.0% isoflurane (Lyppards, Adelaide, South Australia, Australia) (Varcoe et al., 2019). MRI‐compatible SO_2_ and HR were monitored for the ewes for the scan duration (Nonin Medical Inc., Plymouth, MN, USA) and recorded continuously using LabChart 7 (ADInstruments). The ewes were ventilated at a respiratory rate of 16–18 breaths per minute, and 1.0–3.0 l of oxygen was titrated with 3.0–5.0 l of air to match fetal blood gases during MRI to fetal blood gas status before the onset of anaesthesia (baseline). The ewes’ vital signs, including end‐tidal CO_2_, SPO_2_ and HR, were recorded and reviewed every 15 min during the scan to monitor maternal health. Fetal blood pressure waveforms were processed using LabChart 7 to obtain a real‐time cardiac image acquisition trigger for the MRI (Duan et al., 2019, 2017; Schrauben et al., 2019), allowing cine imaging with high spatial and temporal resolution by acquiring data over the cardiac cycle. Briefly high‐resolution steady‐state free precession (SSFP) images of the fetus were collected, and two‐ and four‐chamber balanced SSFP cine slices were obtained. Using the orthogonal long‐axis views, a stack of short‐axis cine slices were obtained, covering the entire heart, as previously published (Cho et al., 2024, 2020). Each MRI session was part of an extensive data collection protocol, and full MRI results will be reported in a separate study. In this study the fetal cardiac function outcomes are correlated with cardiac molecular findings.

### PM and tissue collection

At 139–142dGA pregnant ewes were humanely killed with an intravenous overdose of sodium pentobarbitone (Virbac, Peakhurst, New South Wales, Australia), and fetuses were delivered by hysterectomy (control, *n* = 14 (8m, 6f); LGUN, *n* = 13 (6m, 7f); LGUN+G, *n =* 6 (5m, 1f)). Fetal body, brain and heart weights were recorded. Fetal LV samples were collected and frozen in liquid nitrogen, and a subset was fixed in optimal cutting temperature (OCT) compound and stored at −80°C for subsequent molecular and histological analyses.

### Quantification of gene expression in the left ventricle

#### Total RNA extraction

RNA was extracted and purified from ∼50 mg of fetal LV tissue (control, *n* = 14 (8m, 6f); LGUN, *n* = 13 (6m, 7f); LGUN+G, *n =* 6 (5m, 1f)) using Qiagen QIAzol Lysis Reagent and the RNeasy Mini Kit (Qiagen Pty Ltd Australia, Doncaster, Victoria, Australia) according to the manufacturer's protocol. The purity and concentration of RNA were measured at 260 and 280 nm using a Nanodrop spectrophotometer (Thermo Fisher Scientific, Waltham, Massachusetts, USA), and the integrity of the RNA was determined using agarose gel electrophoresis. cDNA was synthesized using Superscript III (Invitrogen, USA, Carlsbad, California, USA) reverse transcription as previously described (Soo et al., 2012). All samples were run on a no‐amplification control (NAC) plate to ensure RNA samples contained no genomic DNA contamination.

#### Quantitative real‐time PCR

All RT‐PCR procedures adhered to the Minimum Information for Publication of Quantitative Real‐time PCR Experiments (MIQE)B guidelines (Bustin et al., 2009). The expression of the genes of interest included IGF and their receptors (*IGF1*, DQ152962; *IGF1R*, AY162434; *IGF2*, M89789; *IGF2R*, AF327649; Gentili et al., 2009), and cardiac remodelling genes (collagen type 1 alpha 1 chain (*COL1A1*), AF129287; collagen type 3 alpha 1 chain (*COL3A1*), NM_001076831). Tissue inhibitor of metalloproteinases (TIMP) (*TIMP1*, NM_001009319.2; *TIMP2*, NM_001166186; *TIMP3*, NM_001166187.1; Darby et al., 2018; Wang et al., 2013) and metabolism (glucose transporter 4 (*GLUT4*), AB005283; *GLUT1*, U89029.1; peroxisome proliferative activated receptor, gamma, coactivator 1 *(PGC1α)*, BC151326.1; cluster of differential 36 (*CD36)*, BC103112.1 and carnitine palmitoyltransferase 1B (*CPT1β*), NM_001009259.1; Botting et al., 2014; Muhlhausler et al., 2009; Wang et al., 2013) were measured using quantitative RT‐PCR. The expression of these target genes was normalized to three reference genes: hypoxanthine phosphoribosyl transferase (HPRT), ribosomal protein P0 (RPPO) and TATA box binding protein, transcript variant 1 (TBP). Appropriate reference genes were determined as previously described (Lie et al., 2013; McGillick et al., 2013; Soo et al., 2012). Samples were run in triplicate for each target and reference gene, and the mRNA amplification for each sample was determined using KiCqStart SYBR Green qPCR ReadyMix (Sigma‐Aldrich, St. Louis, MO, USA) on a QuantStudio 7 Pro Real‐Time PCR system (Thermo Fisher Scientific). No template controls (NTC) for each primer set were included on each plate to check for non‐specific amplification. The threshold was set within the exponential growth phase of the amplification curve, and the corresponding *Ct* values were obtained to quantify each reaction as previously described (McGillick et al., 2013; Soo et al., 2012).

### Quantification of protein abundance in the left ventricle

#### Protein extraction

In a subset of fetuses (control, *n* = 10 (5m, 5f); LGUN, *n* = 11 (6m, 5f); LGUN+G, *n =* 6 (5m, 1f)), LV tissue (∼100 mg) was sonicated (3 × 15‐s burst at 50% amplitude; John Morris Scientific, SA, Australia) in 1 ml lysis buffer consisting of 1 mm Tris–HCl (pH = 8), 5 m NaCl, 1% NP‐40, 1mm sodium orthovanadate, 30 mM NaF, 10 mM sodium tetrapyrophosphate, 10 mM EDTA and a protease inhibitor tablet consisting of 3.7 mg EDTA/tablet (complete Mini, Roche, Indianapolis, IN, USA). Samples were then centrifuged at 14,300 *g* and 4°C for 14 min (Eppendorf Centrifuge 5415, Crown Scientific, Macquarie Park, Victoria, Australia), the supernatant was collected into new 1.5 ml Eppendorf tubes and protein concentration was quantified using a Micro BCA Protein Assay Kit as previously described (Botting et al., 2018, 2014; Darby et al., 2018; Lie et al., 2013; Soo et al., 2012).

#### Western blotting

Each sample (75 µg) was subjected to SDS‐PAGE set at a constant 40 V and then transferred at 800 mA onto a nitrocellulose membrane (Hybond ECL, GE Healthcare, Mascot, New South Wales, Australia). Membranes were subsequently stained with Ponceau S (0.5% Ponceau in 1% acetic acid) to quantify the total protein on the membrane for normalization. Images of the membranes were obtained using ImageQuant LAS4000 (GE Healthcare, Chicago, IL, USA), and total protein was quantified by densitometry using ImageQuant analysis software (GE Healthcare, version 8.1, GE Healthcare, Chicago, IL, USA) as previously described (Darby et al., 2018; Soo et al., 2012). The membranes were then subjected to three 5‐min washes in Tris‐buffered saline (TBS), cut according to the size of the proteins and then blocked in 5% bovine serum albumin (BSA) in TBS with 1% Tween (TBST‐T) for 1 h at room temperature with agitation. The membranes underwent three 5‐min washes in TBST‐T with agitation and were incubated with their respective primary antibody in 5% BSA/TBST‐T overnight at 4°C. Primary antibodies of interest were mitobiogenesis (1:250, ab123545, Abcam), total OXPHOS (1:1000, ab110413, Abcam; Darby, et al., 2020), GLUT4 (1:1000, ab33780, Abcam; Wang et al., 2013), glycogen synthase kinase 3 alpha/beta (GSK3α/β, 1:1000, SC‐7291, Santa‐Cruz), phosphorylated glycogen synthase kinase α (pGSK3α, 1:1000, 9316S, Cell Signaling Technology), phosphorylated glycogen synthase kinase β (pGSK3β, 1:1000, sc‐11757, Santa‐Cruz), glycogen synthase (GS, 1:1000, 3886S, Cell Signaling Technology), phosphorylated glycogen synthase (pGS, 1:1000, 3891S, Cell Signaling Technology; Wang et al., 2013, 2011, 2015), optic atrophy 1 (OPA1, 1:1000, 80471, Cell Signaling Technology), dynamin‐related protein 1 (DRP1, 1:1000, 8570, Cell Signaling Technology), mitofusin‐2 (MFN2, 1:1000, 9482, Cell Signaling Technology; Bertossa et al., 2024), Ca^2+^/calmodulin‐dependent protein kinase II (CAMKII, 1:1000, 3362, Cell Signaling Technology; Darby et al., 2018; Wang et al., 2015, 2011), phosphorylated CAMKII (p‐CAMKII, sc‐32289, Abcam), phospholamban (PLN, 1:1000, 8495, Cell Signaling Technology), sarcoplasmic/endoplasmic reticulum Ca^2+^‐ATPase (SERCA, 1:1000, ab2861, Abcam) and troponin I (1:1000, 4004, Cell Signaling Technology; Darby et al., 2018). After incubation with the primary antibody, the membranes were subjected to three 5‐min washes in TBST‐T and incubated with relevant HRP‐labelled secondary antibodies: rabbit (1:2000, 7074, Cell Signaling Technology) and mouse (1:2000, 7077, Cell Signaling Technology) for 1 h at room temperature with agitation. Enhanced chemiluminescence was used to detect reactive bands using SuperSignal West Pico Chemiluminescent Substrate (Thermo Scientific). The image of the membrane was obtained using ImageQuant LAS4000 (GE Healthcare, Chicago, IL, USA), and protein abundance was quantified by densitometry using ImageQuant analysis software (GE Healthcare, Chicago, IL, USA) as previously described (Darby et al., 2018; Wang et al., 2013, 2011, 2015).

### Quantification of hormone concentrations in fetal heart tissue

Fetal LV hormone concentrations (control, *n* = 14 (8m, 6f); LGUN, *n =* 13 (6m, 7f); LGUN+G, *n =* 6 (5m, 1f)) were determined using liquid chromatography (LC, Shimadzu Nexera XR, Shimadzu, Kyoto, Japan) coupled to a Sciex 6500 Triple‐Quad system (MS/MS, Sciex, Framingham, MA, USA) using an adapted protocol (Lock et al., 2023; McBride et al., 2020). Heart tissue homogenates (prepared in 0.9% NaCl) were subjected to liquid–liquid extraction using acetonitrile and ethyl acetate as previously described (Bertossa et al., 2024; Dimasi, Darby, et al., 2023, Dimasi et al., 2024; Meakin et al., 2024). Prepared samples were injected onto an Acquity UPLC BEH C18 column (130 Å, 1.7 µm, 2.1 × 100 mm, Waters Corp., Massachusetts, USA). Hormone concentrations were calculated via integration with a standard curve that ranged from 0.05 to 100 ng/ml. Data are presented and indexed based on heart tissue weight.

### Enzymatic activity assay

A lactate dehydrogenase (LDH) assay kit (ab102526, Abcam, Cambridge, UK) and citrate synthase (CS) assay kit (CS0720, Sigma‐Aldrich, St. Louis, USA) were used to quantify the enzymatic activities of LDH and CS, respectively, in fetal LV tissue (∼100 mg; control, *n* = 14 (8m, 6f); LGUN, *n =* 13 (6m, 7f); LGUN+G, *n =* 6 (5m, 1f)). The assays were performed according to the manufacturer's protocol and are previously described in detail (Dimasi et al., 2024).

### Periodic acid–Schiff and Masson's trichrome staining and quantification in fetal heart tissue

In a subset of fetuses (control, *n* = 13 (7m, 6f); LGUN, *n* = 9 (3m, 6f); LGUN+G, *n =* 6 (5m, 1f)), LV tissue samples embedded in OCT were sectioned (5 µm, cryostat, Adelaide, Australi) onto super‐frost slides (VWR International, Randor, PA, USA). The slides were then stained with periodic acid–Schiff (PAS) or Masson's trichrome by a service provider (Adelaide University Histology Services); LV slides were then scanned at 40× magnification using a NanoZoomer‐XR (Hamamatsu, Japan) to obtain whole‐slide images. Glycogen content was determined from PAS‐stained slides analysed in Fiji/Image J software version 1.54f (version 1.54f, NIH, Bethesda, MD, USA) using colour threshold methods at 20× magnification (five frames 1 mm apart). Collagen content was determined from Masson's trichome‐stained slides analysed using Visiopharm software version 2020.8 (Visiopharm 2020.08, Horsholm, Denmark). Briefly the total area of collagen staining was measured using a custom threshold analysis at 10× magnification, progressively covering the entire surface of the tissue section. The threshold included two masks, one for tissue background staining and one for collagen (blue), as previously described (Zhang et al., 2024). Correct quantification of the staining was confirmed by visual examination by a trained individual blinded to the treatment groups as previously described (Lock et al., 2019).

### Statistical analysis

Data are presented as mean ± SD, and *P* ≤ 0.05 was considered significant for all analyses. Normality was assessed using a Shapiro–Wilk test where *P* < 0.05 indicated non‐normality. Due to limited tissue availability and thus unbalanced sex distribution between groups, no attempt was made to analyse data via two‐way ANOVA; however fetal sex is indicated in figures by different symbols. All data were normally distributed; thus one‐way ANOVA followed by a *post hoc* Bonferroni multiple comparisons test was used to determine the effects of LGUN and LGUN+G on maternal and fetal characteristics, hormone, gene and protein measures. Simple linear regression was used to determine the relationship between mean fetal gestational plasma glucose and cardiac function parameters with hormone concentrations, gene expression and protein abundance. Outliers were identified using Grubbs's test (*P* < 0.05) and excluded (GraphPad Prism version 10.0.2 for Windows, GraphPad Software, La Jolla, CA, USA).

## Results

### Maternal outcome

There was no difference in maternal weight between treatment groups on the day of allocation or the day of MRI (Table 1); however maternal weight change between these time points was significantly different in LGUN and LGUN+G compared to that of controls (*P* = 0.0145; Table 1). During the treatment period LGUN and LGUN+G had no impact on mean gestational maternal plasma glucose between treatment groups (Table 1).

**Table 1:** The impact of LGUN and LGUN+G on maternal and fetal characteristics.

	Control (*n* = 14, 8m, 6f)	LGUN (*n* = 13, 6m, 7f)	LGUN+G (*n* = 6, 5m, 1f)	*p‐*Value
Maternal outcomes				
Maternal weight at allocation (∼111dGA) (kg)	60.71 (3.04)	63.73 (6.79)	66.83 (5.02)	0.0571
Maternal weight at MRI (∼139–142dGA) (kg)	63.36 (4.90)	62.65 (6.82)	64.75 (3.66)	0.7512
Maternal weight change across dietary protocol (kg)	2.64 (3.39)^a^	−1.08 (4.58)**^b^**	−2.1 (1.72)**^b^**	**0.0145**
Maternal mean gestational blood glucose concentration (mmol/l)	2.05 (0.25)	1.69 (0.16)	2.01 (0.47)	0.0863
Mean gestational fetal blood gas measures				
PaO_2_ (mmHg)	18.8 (1.6)	18.6 (1.9)	19.0 (0.8)	0.847
PaCO_2_ (mmHg)	50.4 (1.9)	51.0 (2.6)	50.8 (1.5)	0.744
pH	7.361 (0.012)^a^	7.375 (0.011)**^b^**	7.388 (0.014)**^b^**	0.0003
Lactate (mmol l^−1^)	1.2 (0.2)	1.2 (0.2)	1.4 (0.2)	0.347
Hb (g/dl)	105 (21)	98 (8)	100 (4)	0.564
Hct (%)	30.8 (6.2)	29.1 (1.9)	29.4 (1.3)	0.364
SaO_2_ (%)	55.6 (7.9)	59.0 (6.2)	61.0 (4.2)	0.232
Fetal heart weight at postmortem				
Heart weight (g)	32.69 (6.28)	30.48 (5.53)	30.19 (4.91)	0.853
Heart weight:body weight (g kg^−1^)	6.47 (0.74)	6.48 (0.58)	6.12 (0.42)	0.394
Left ventricle weight (mg)	10.86 (2.29)	9.93 (1.77)	9.68 (2.28)	0.686
Left ventricle:heart weight (mg g^−1^)	0.33 (0.03)	0.33 (0.03)	0.32 (0.04)	0.641
Right ventricle weight (mg)	9.69 (1.90)	9.05 (1.75)	8.56 (1.33)	0.410
Right ventricle:heart weight (mg g^−1^)	0.30 (0.03)	0.30 (0.04)	0.28 (0.02)	0.628

Data are expressed as mean (SD) and analysed using one‐way ANOVA followed by a *post hoc* Bonferroni multiple comparisons test. ns, *P* > 0.05. Superscript alphabets indicate significant differences between treatment groups (*P* < 0.05) such that values with different alphabets letters are statistically different from each other, and values with the same alphabetical letters are not different.

dGA, days gestational age; f, female; Hb, haemoglobin; Hct, haematocrit; LGUN, late‐gestation undernutrition; LGUN+G, late‐gestation undernutrition + glucose; m, male; *n*, number of animals; PaCO_2_, partial pressure of carbon dioxide; PaO_2_, partial pressure of oxygen; pH, blood acidity/basicity; SaO_2_, oxygen saturation.

### Fetal outcomes

**Figure 1:**
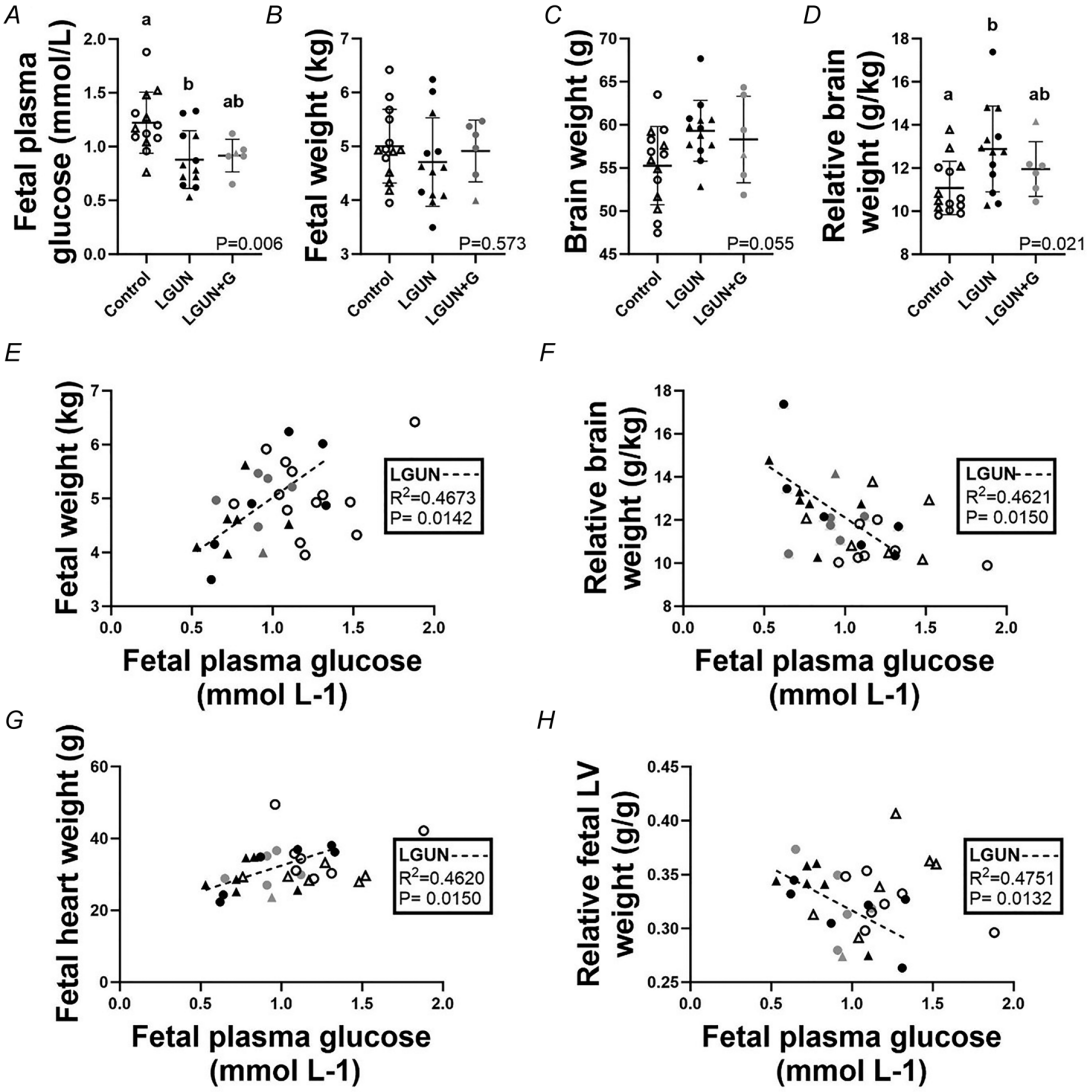
The impact of LGUN and LGUN+G on fetal characteristics The impact of LGUN (late‐gestation undernutrition) and LGUN+G (late‐gestation undernutrition + glucose) on mean gestational fetal plasma glucose concentration (*A*), fetal body weight (*B*), fetal brain weight (*C*) and brain weight relative to total body weight (*D*) recorded on the day of postmortem. The relationship between fetal plasma glucose and fetal body weight (*E*), brain weight relative to total body weight (*F*), fetal heart weight (*G*) and left ventricle weight relative to heart weight (*H*) in the LGUN group only. Circles, males (m); triangles, females (f). Controls in open data points (*n* = 14, 8m, 6f), LGUN in black data points (*n* = 13, 6m, 7f) and LGUN+G in grey data points (*n* = 6, 5m, 1f). Data are expressed as mean ± SD and analysed using one‐way ANOVA followed by a *post hoc* Bonferroni multiple comparisons test. ns, *P* > 0.05. Superscript alphabets indicate significant differences between treatment groups (*P* < 0.05) such that values with different alphabets are statistically different from each other and values with the same alphabets are not different.

LGUN and LGUN+G had no impact on mean gestational fetal blood gas measurements of arterial *P*O_2_, *P*CO_2_, Hb or lactate (Table 1). LGUN and LGUN+G increased mean gestational pH compared to that of controls; however values were in physiologically normal ranges (Table 1). LGUN and LGUN+G had no impact on mean basal SBP, DBP, MAP or HR at 118–119 or 137–1388dGA (Table 2). LGUN reduced mean fetal plasma glucose concentration compared to that of controls (Fig. 1*A*; *P* = 0.0095). LGUN and LGUN+G had no impact on fetal body weight (Fig. 1*B*) or fetal brain weight (Fig. 1*C*) measured on the day of PM; however brain weight relative to fetal body weight was increased in LGUN compared to controls (Fig. 1*D*; *P* = 0.0158). There was a positive relationship between fetal weight and fetal plasma glucose concentrations in LGUN (*R* ^2^ = 0.4673, *P* = 0.0142; Fig. 1*E*) and a negative relationship between relative brain weight and fetal plasma glucose concentrations in LGUN (*R* ^2^ = 0.4621, *P* = 0.0150; Fig. 1*F*), but these relationships were absent in controls and LGUN+G when data were stratified by treatment.

**Table 2:** The impact of LGUN and LGUN+G on basal fetal blood pressure between 118–119 and 137–138dGA.

Basal blood pressure at 118–119dGA	Control (*n* = 10, 6m, 4f)	LGUN (*n* = 8, 4m, 4f)	LGUN+G (*n* = 6, 5m, 1f)	*p‐*Value
SBP (mmHg)	43 (4)	47 (5)	46 (10)	0.194
DBP (mmHg)	29 (5)	31 (4)	33 (9)	0.586
MAP (mmHg)	33 (6)	38 (3)	38 (9)	0.329
HR (bpm)	178 (11)	170 (9)	177 (5)	0.183
Basal blood pressure at 137–1388dGA	Control (*n* = 8, 4m, 4f)	LGUN (*n* = 6, 2m, 4f)	LGUN+G (*n* = 5, 5m, 0f)	*p‐*Value
SBP (mmHg)	56 (9)	53 (4)	55 (5)	0.700
DBP (mmHg)	38 (6)	33 (4)	36 (4)	0.171
MAP (mmHg)	45 (6)	41 (3)	43 (4)	0.285
HR (bpm)	148 (13)	145 (20)	143 (6)	0.856

Data are expressed as mean (SD) and analysed using one‐way ANOVA followed by a *post hoc* Bonferroni multiple comparisons test. ns, *P* > 0.05. Exclusion criteria for blood pressure study included catheter not present at 118dGA (control *n* = 2) or 138dGA (control *n* = 3, LGUN *n* = 1, LGUN + G *n* = 1), poor pulsatility at 118dGA (control *n* = 2, LGUN *n* = 3) or 138dGA (control *n* = 3, LGUN *n* = 3) and blood pressure study not performed at 118dGA (control *n* = 2, LGUN *n* = 3) or 138dGA (control *n* = 3, LGUN *n* = 3).

DBP, diastolic blood pressure; dGA, days gestational age; f, female; HR, heart rate; m, male; LGUN, late‐gestation undernutrition; LGUN+G, late‐gestation undernutrition + glucose; MAP, mean arterial pressure; *n*, number of animals; SBP, systolic blood pressure.

### The impact of LGUN and LGUN+G on fetal heart development

LGUN and LGUN+G had no impact on absolute or relative heart or ventricle weight measured on the day of PM (Table 1). There was a positive relationship between absolute heart weight and fetal plasma glucose (*R* ^2^ = 0.4620, *P* = 0.0150; Fig. 1*G*) and a negative relationship between LV relative to fetal weight and fetal plasma glucose in LGUN (*R* ^2^ = 0.4751, *P* = 0.0132; Fig. 1*H*), but these relationships were absent in controls and LGUN+G when data were stratified by treatment. LGUN+G increased the mRNA expression of *IGF1* compared to controls and LGUN (*P* = 0.0159; Table 3). LGUN and LGUN+G had no impact on the mRNA expression of *IGF1R*, *IGF2*, *IGF2R, MIES1, Ki‐67* or *PCNA* (Table 3).

**Table 3:** The impact of LGUN and LGUN+G on the mRNA and protein expression and enzyme activity of factors regulating growth and metabolism in the fetal LV.

Gene (MNE)	Control (*n* = 8m, 6f)	LGUN (*n* = 6m, 7f)	LGUN+G (*n* = 5m, 1f)	*p‐*Value
*IGF1*	0.446 (0.147)^a^	0.473 (0.299)^a^	0.917 (0.608)^b^	0.0159
*IGF1R*	1.285 (0.308)	1.232 (0.304)	1.111 (0.306)	0.516
*IGF2*	26.642 (6.91)	24.621 (7.028)	23.659 (13.31)	0.715
*IGF2R*	2.932 (0.482)	2.523 (0.42)	2.875 (0.473)	0.070
*GLUT1*	0.053 (0.005)	0.057 (0.005)	0.048 (0.007)	0.260
*GLUT4*	0.803 (0.131)	0.682 (0.207)	0.692 (0.204)	0.066
*CD36*	17.921 (3.095)	14.656 (1.332)	13.633 (2.588)	0.722
*CPT1β*	1.088 (0.383)^a^	0.648 (0.060)^ab^	0.445 (0.079)^b^	0.022
*PGC1α*	1.284 (0.406)	0.856 (0.073)	0.765 (0.081)	0.421
*MIES1*	0.068 (0.011)	0.059 (0.020)	0.057 (0.015)	0.233
*Ki‐67*	0.066 (0.034)	0.069 (0.042)	0.072 (0.035	0.950
*PCNA*	0.244 (0.065)	0.212 (0.031)	0.270 (0.093)	0.172
*DIO1*	0.003 (0.002)	0.007 (0.008)	0.066 (0.07)	0.0009
Protein (AU)	Control (*n* = 5m, 5f)	LGUN (*n* = 6m, 5f)	LGUN+G (*n* = 5m, 1f)	*p*‐Value
GLUT4: Ponceau S	0.021 (0.009)^a^	0.010 (0.003)^b^	0.007 (0.004)^b^	0.0001
OPA1: Ponceau S	0.0001 (0.001)	0.0008 (0.0005)	0.0009 (0.0006)	0.847
DRP1: Ponceau S	0.009 (0.005)	0.009 (0.004)	0.010 (0.006)	0.660
MFN2: Ponceau S	0.003 (0.003)^a^	0.004 (0.004)^a^	0.010 (0.007)^b^	0.016
SERCA: Ponceau S	0.11081 (0.03992)	1.3592 (0.04303)	0.13777 (0.05546)	0.364
Troponin: βtubulin	1.145 (0.943)	0.825 (0.466)	0.786 (0.408)	0.162
PLN: βtubulin	2.914 (2.361)	6.0167 (6.0227)	3.820 (2.120)	0.278
Enzyme activity	Control (*n* = 8m, 6f)	LGUN (*n* = 6m, 7f)	LGUN+G (*n* = 5m, 1f)	*p*‐Value
Lactate dehydrogenase activity	8712 (2514)	8619 (2521)	8194 (2255)	0.922
Citrate synthase activity	2.10 (0.56)	2.48 (0.55)	2.68 (0.46)	0.065

Data are expressed as mean (SD) and analysed using one‐way ANOVA followed by a *post hoc* Bonferroni multiple comparisons test. ns, *P* > 0.05. Superscript alphabets indicate significant differences between treatment groups (*P* < 0.05) such that values with different alphabets are statistically different and those with the same alphabets are not different.

AU, arbitrary units; DRP1, dynamin‐related protein 1; f, female; GLUT4, glucose transporter 4; IGF, insulin‐like growth factor; LGUN, late‐gestation undernutrition; LGUN+G, late‐gestation undernutrition + glucose; LV, left ventricle; m, male; MNE, mean normalized expression; *n*, number of animals; OPA1, optic atrophy 1; PLN, phospholamban; SERCA, sarcoplasmic/endoplasmic reticulum Ca^2+^‐ATPase.

### Glucose infusion into the LGUN fetus increased the concentration of T4 and *DIO1* gene expression in the fetal heart

**Figure 2:**
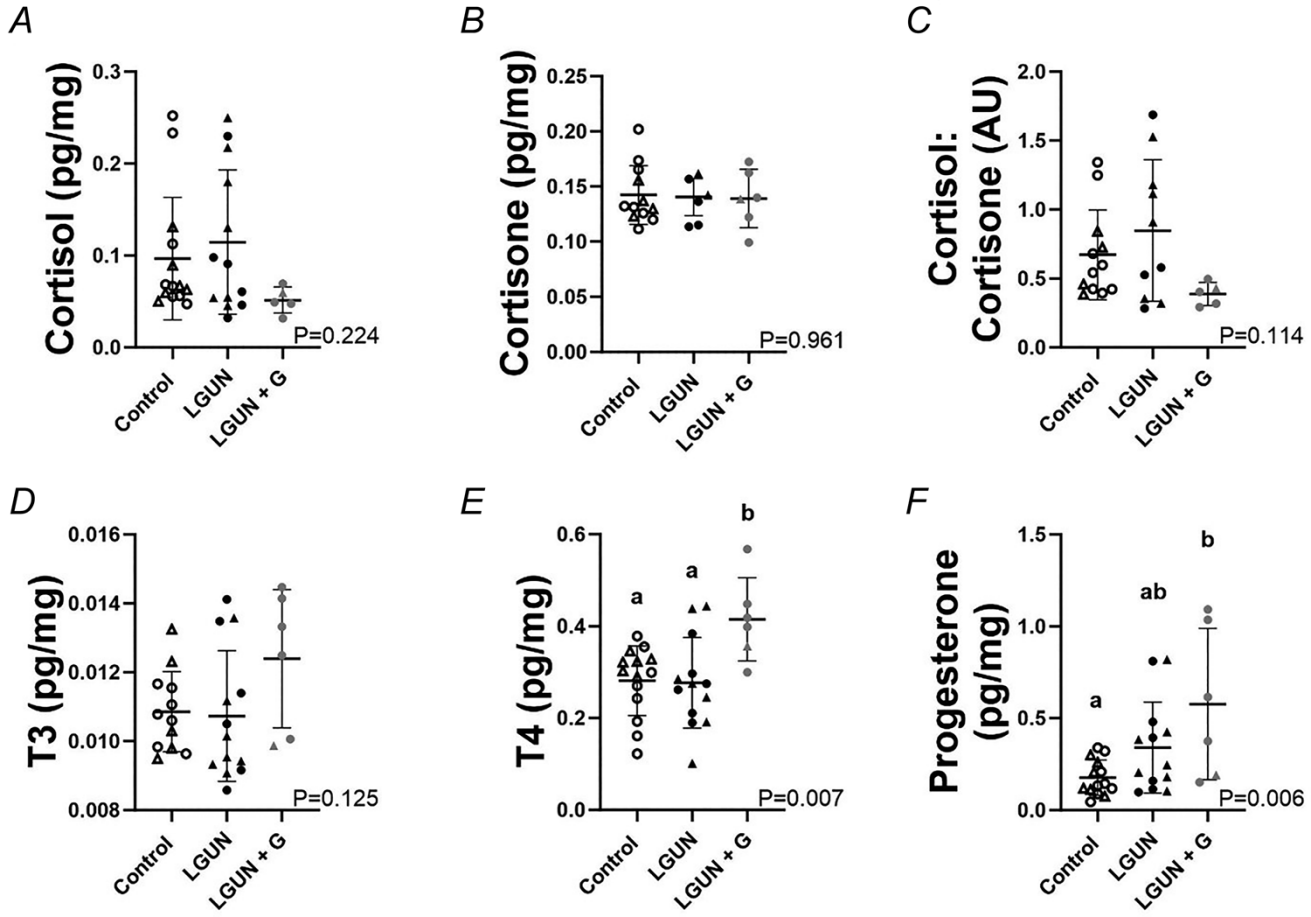
The impact of LGUN and LGUN+G on fetal cardiac hormone concentrations The impact of LGUN (late‐gestation undernutrition) and LGUN+G (late‐gestation undernutrition + glucose) on cardiac tissue‐specific glucocorticoid concentrations of cortisol (*A*), cortisone (*B*), the cortisol:cortisone ratio (*C*), T3 (*D*), T4 (*E*) and progesterone (*F*). Circles, males (m); triangles, females (f). Controls in open data points (*n* = 14, 8m, 6f), LGUN in black data points (*n* = 13, 6m, 7f) and LGUN+G in grey data points (*n* = 6, 5m, 1f). Data are expressed as mean ± SD and analysed using one‐way ANOVA followed by a *post hoc* Bonferroni multiple comparisons test. ns, *P* > 0.05. Superscript alphabets indicate significant differences between treatment groups (*P *< 0.05) such that values with different alphabets are statistically different and those with the same alphabets are not different.

LGUN and LGUN+G had no impact on cardiac tissue‐specific concentrations of cortisol (Fig. 2*A*), cortisone (Fig. 2*B*) or the ratio of cortisol to cortisone (Fig. 2*C*). LGUN and LGUN+G had no impact on T3 concentrations between treatment groups (Fig. 2*D*); however T4 concentrations were increased in LGUN+G compared to controls and LGUN (Fig. 2*E*; *P* = 0.0121 and 0.0103, respectively). Progesterone concentrations were increased in LGUN+G compared to controls but were not different from LGUN (Fig. 2*F*, *P* = 0.0051). *DIO1* gene expression was increased in LGUN+G compared to controls and LGUN (Table 3; *P* = 0.0013 and 0.0023, respectively).

### Neither LGUN nor glucose infusion had an impact on markers of fibrosis or area of fibrosis staining in the fetal LV

**Figure 3:**
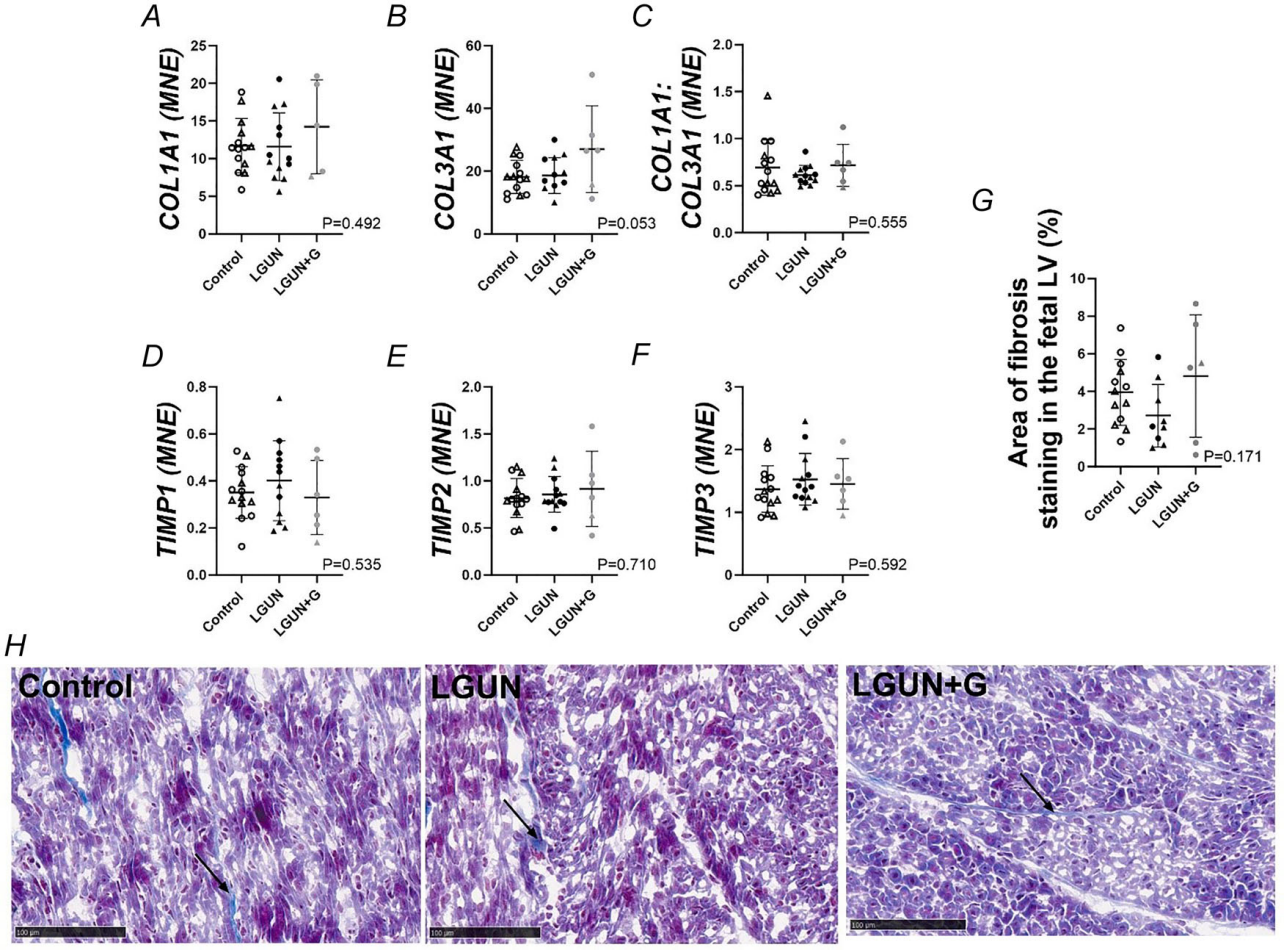
The impact of LGUN and LGUN+G on markers of remodelling in the fetal heart The impact of LGUN (late‐gestation undernutrition) and LGUN+G (late‐gestation undernutrition + glucose) on the cardiac mRNA expression of COL1A1 (*A*), COL3A1 (*B*), COL1A1:COL3A1 (*C*), TIMP1 (*D*), TIMP2 (*E*) and TIMP3 (*F*). Area of fibrosis staining in the fetal LV (left ventricle) (*G*). Representative microimages (scale bar = 100 µm, 40× magnification) of Masson's trichrome staining for each treatment group, with arrows indicating collagen stained in blue (*H*). Circles, males (m); triangles, females (f), Controls in open data points (gene: *n* = 14, 8m, 6f; histology: *n* = 13, 7m, 6f), LGUN in black data points (gene: *n* = 13, 6m, 7f; histology: *n* = 9, 3m, 6f) and LGUN+G in grey data points (gene: *n* = 6, 5m, 1f; histology: *n* = 6, 5m, 1f). Data are expressed as mean ± SD and analysed using one‐way ANOVA followed by a *post hoc* Bonferroni multiple comparisons test. ns, *P* > 0.05.

LGUN and LGUN+G had no impact on the mRNA expression of *COL1A1* (Fig. 3*A*), *COL3A1* (Fig. 3*B*), the ratio of *COL1A1* to *COL3A1* (Fig. 3*C*), *TIMP1* (Fig. 3*D*), *TIMP2* (Fig. 3*E*) or *TIMP3* (Fig. 3*F*). LGUN and LGUN+G had no impact on the area of fibrosis staining in the fetal LV (Fig. 3*G*).

### LGUN reduces the protein abundance of OXPHOS complex 3 and is restored by glucose infusion in the fetal LV

**Figure 4:**
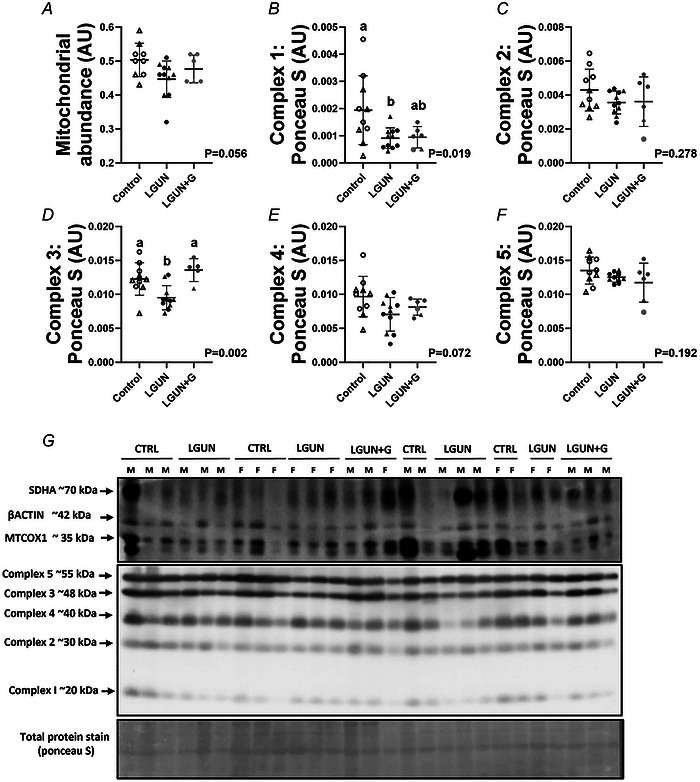
The impact of LGUN and LGUN+G on mitochondrial abundance and mitochondrial oxidative phosphorylation complexes in the fetal heart The impact of LGUN (late‐gestation undernutrition) and LGUN +G (late‐gestation undernutrition + glucose) on cardiac mitochondrial abundance (*A*) oxidative phosphorylation complex 1 (*B*), complex 2 (*C*), complex 3 (*D*), complex 4 (*E*) and complex 5 (*F*). Data are presented as normalized protein expression in arbitrary units (AU). Circles, males (m); triangles, females (f). Controls in open data points (*n* = 10, 5m, 5f), LGUN in black data points (*n* = 11, 6m, 5f) and LGUN+G in grey data points (*n* = 6, 5m, 1f). Western blot images represent the target protein and the total protein stain (Ponceau S) used for protein normalization for controls (CTRL), LGUN and LGUN+G (*G*). Data are expressed as mean ± SD and analysed using one‐way ANOVA followed by a *post hoc* Bonferroni multiple comparisons test. ns, *P* > 0.05. Superscript alphabets indicate significant differences between treatment groups (*P* < 0.05) such that values with different alphabets are statistically different and those with the same alphabets are not different.

LGUN or LGUN+G had no impact on mitochondrial abundance (Fig. 4*A*) determined by the ratio of mitochondria DNA‐encoded COX‐I (SDHA) to nuclear DNA‐encoded COXII (MTCOXI). LGUN decreased the protein abundance of OXPHOS complex 1 compared to controls (Fig. 4*B*; *P* = 0.0278). Neither LGUN nor LGUN+G had an impact on the abundance of OXPHOS complex 2 (Fig. 4*C*). LGUN decreased the protein abundance of OXPHOS complex 3 compared to controls and LGUN+G (Fig. 4*D*; *P* = 0.0149 and 0.0034, respectively). LGUN and LGUN+G had no impact on the abundance of OXPHOS complexes 4 and 5 (Fig. 4*E* and *F*).

### Glucose infusion into the LGUN fetus alters markers of mitochondrial fusion and fatty acid transport in the fetal LV

LGUN or LGUN+G did not impact CS activity, the mRNA expression of PGC1α or the protein abundance of OPA1 and DRP1 (Table 3). LGUN+G increased the protein abundance of MFN2 compared to controls and LGUN (*P* = 0.0161; Table 3). LGUN+G decreased the mRNA expression of fatty acid transporter CPT1β compared to that of controls but not LGUN+G (*P* = 0.0223; Table 3); however there was no impact on cellular fatty acid transporter CD36 (Table 3). Protein abundance of GLUT4 was reduced in both LGUN and LGUN+G compared to controls (*P* = 0.0010 and 0.0003, respectively; Table 3). LGUN and LGUN+G did not impact the mRNA expression of *GLUT4, GLUT1* or LDH enzyme activity (Table 3).

### Glucose infusion into the LGUN fetus alters markers of glycogen synthesis and decreases the percentage of glycogen in the fetal LV

**Figure 5:**
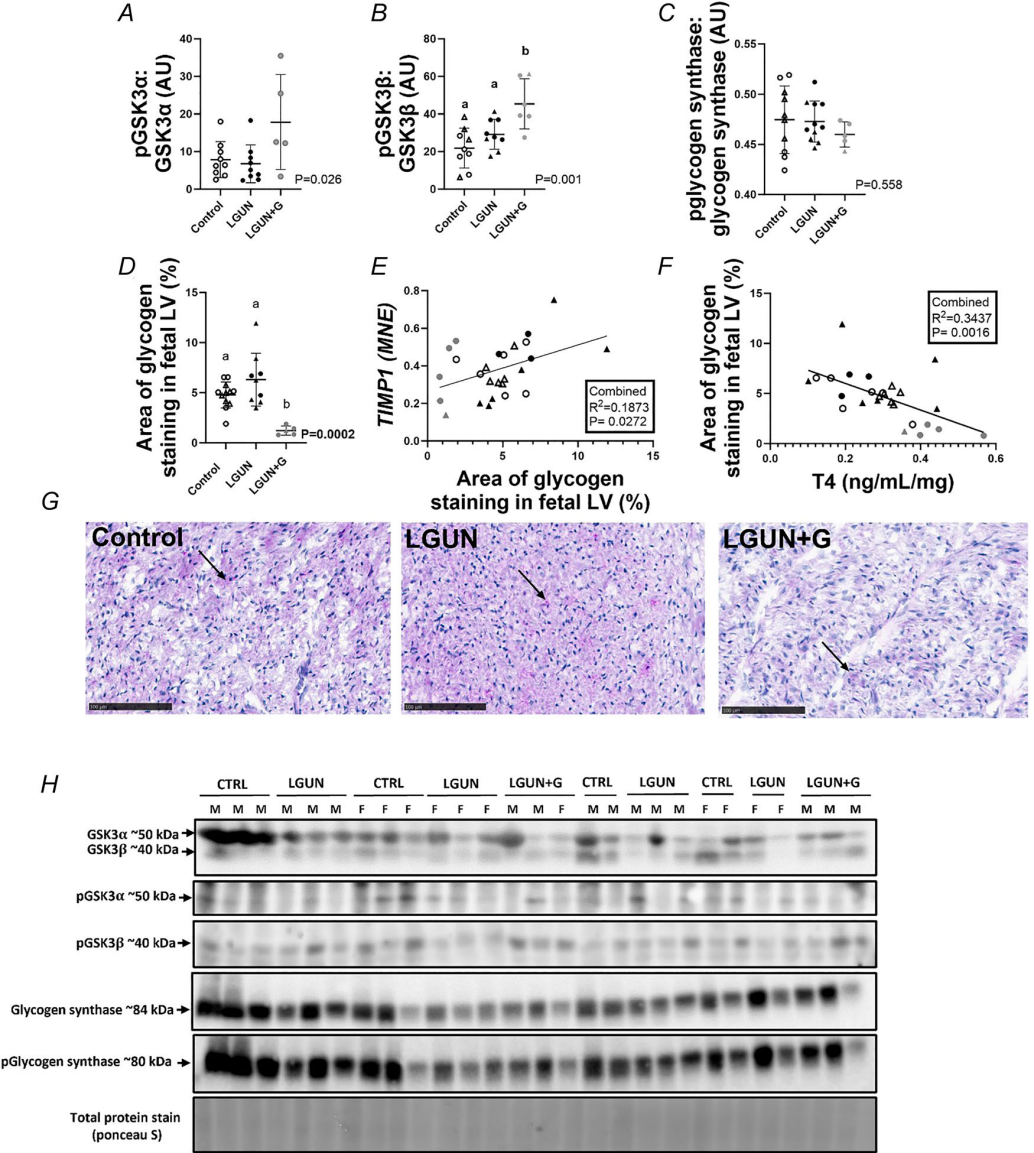
The impact of LGUN and LGUN+G on markers of cardiac glycogen metabolism in the fetal heart The impact of LGUN (late‐gestation undernutrition) and LGUN+G (late‐gestation undernutrition + glucose) on the protein abundance of pGSK3α:GSK3α (phosphorylated glycogen synthase kinase α) (*A*), pGSK3β:GSK3β (phosphorylated glycogen synthase kinase β) (*B*), pglycogen synthase:glycogen synthase (*C*), area of glycogen staining (*D*) and the relationship between the area of glycogen staining and *TIMP1* (*E*) and T4 concentrations (*F*). Representative microimages (scale bar = 100 µm, 40× magnification) of PAS (periodic acid–Schiff) staining for each treatment group, with arrows indicating glycogen stained in magenta (*G*). Data are presented as normalized protein expression in arbitrary units (AU). Circles, males (m); triangles, females (f). Controls in open data points (protein: *n* = 10, 5m, 5f; histology: *n* = 13, 7m, 6f), LGUN in black data points (protein: *n* = 11, 6m, 5f; histology: *n* = 9, 3m, 6f) and LGUN+G in grey data points (protein: *n* = 6, 5m, 1f; histology: *n* = 6, 5m, 1f). Western blot images represent the target protein and the total protein stain (Ponceau S; representative image shown) used for protein normalization for controls (CTRL), LGUN and LGUN+G (*H*). Data are expressed as mean ± SD and analysed using one‐way ANOVA followed by a *post hoc* Bonferroni multiple comparisons test. ns, *P* > 0.05. Superscript alphabets indicate significant differences between treatment groups (*P* < 0.05) such that values with different alphabetical letters are statistically different and values with the same alphabets are not different.

LGUN+G increased the phosphorylation of GSK3α compared to LGUN (pGSK3α:GSK3α; Fig. 5*A*; *P* = 0.0355) and GSK3β compared to controls and LGUN (pGSK3β:GSK3β; Fig. 5*B*; *P* = 0.0010 and 0.0236, respectively). Neither LGUN nor LGUN+G had an impact on the phosphorylation of GS (Fig. 5*C*). LGUN+G decreased the area of glycogen staining compared to that of both controls and LGUN (Fig. 5*D*; *P* = 0.0037 and 0.001, respectively), and this was positively correlated with the mRNA expression of *TIMP1* (*R* ^2^ = 0.1873, *P* = 0.0272; Fig. 5*E*) and negatively correlated with T4 concentrations (*R* ^2^ = 0.3431, *P* = 0.0017; Fig. 5*F*) across all groups.

### Increased activation of CAMKII by LGUN is restored by intrafetal glucose infusion in the fetal LV

**Figure 6:**
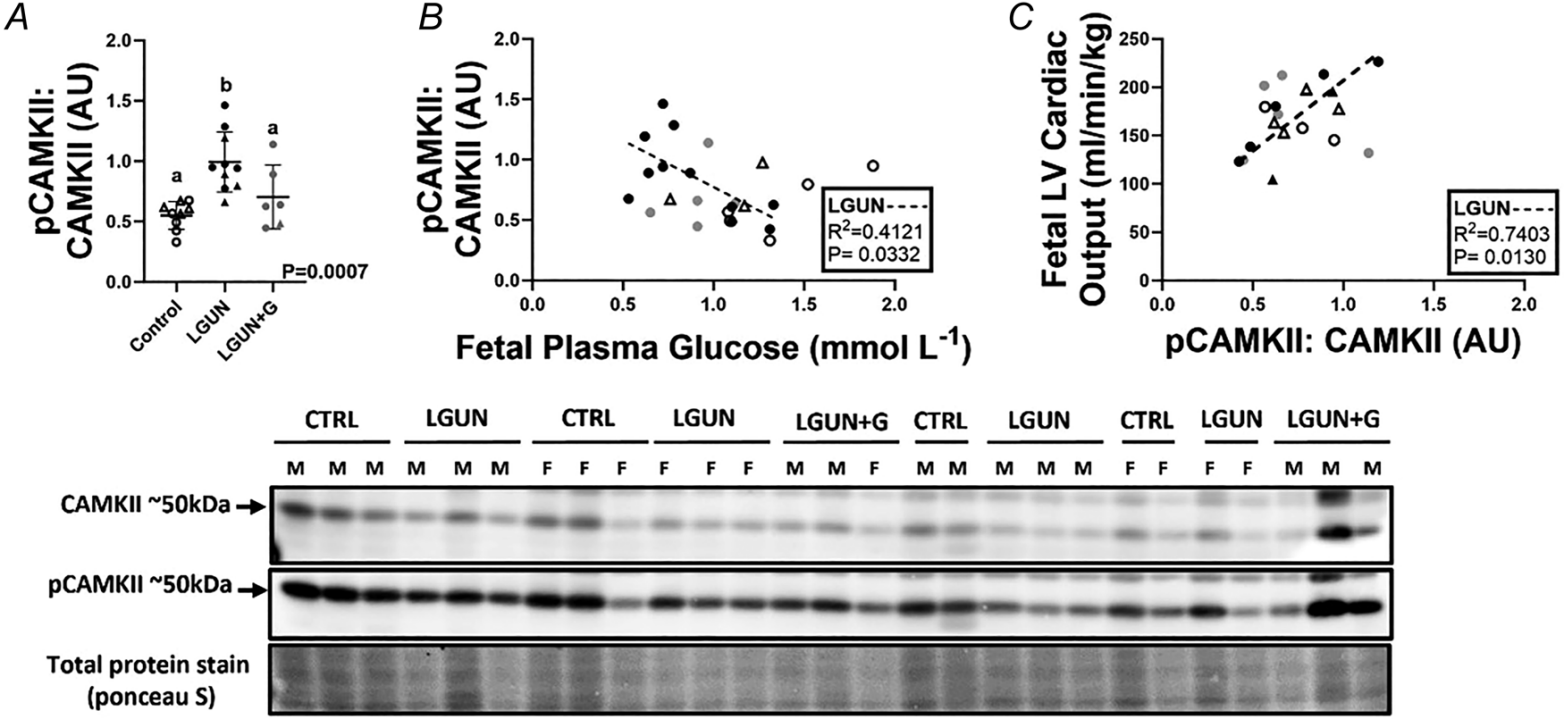
The impact of LGUN and LGUN+G on markers of fetal cardiac contractility The impact of LGUN (late‐gestation undernutrition) and LGUN+G (late‐gestation undernutrition + glucose) on the protein abundance of cardiac p‐CAMKII:CAMKII (*A*); the relationship between p‐CAMKII and fetal plasma glucose in all groups (*B*); the relationship between p‐CAMKII:CAMKII and fetal LV (left ventricle) cardiac output only in LGUN (*C*). Data are presented as normalized protein expression in arbitrary units (AU). Circles, males (m); triangles, females (f). Controls in open data points (protein: *n* = 10, 5m, 5f), LGUN in black data points (*n* = 11, 6m, 5f) and LGUN+G in grey data points (*n* = 6, 5m, 1f). Western blot images represent the target protein and reference protein for controls (CTRL), LGUN and LGUN+G (D). Data are expressed as mean ± SD and analysed using one‐way ANOVA followed by a *post hoc* Bonferroni multiple comparisons test. ns, *P* > 0.05. Superscript alphabets indicate significant differences between treatment groups (*P* < 0.05) such that values with different alphabets are statistically different and those with the same alphabets are not different.

LGUN and LGUN+G had no impact on the protein abundance of troponin I, PLN or SERCA (Table 3). LGUN increased the protein abundance of phosphorylated CAMKII relative to total CAMKII compared to controls and LGUN+G (p‐CAMKII:CAMKII; *P* = 0.0005 and 0.0490, respectively; Fig. 6*A*). There was a negative relationship between p‐CAMKII and fetal plasma glucose concentrations in LGUN (*R* ^2^ = 0.4600, *P* = 0.0218; Fig. 6*B*) that was not present in controls or LGUN+G when data were stratified by treatment. There was a positive relationship between p‐CAMKII and cardiac output relative to fetal weight in the LGUN group (*R* ^2^ = 0.7403, *P* = 0.0130; Fig. 6*C*) that was not present in controls or LGUN+G when data were stratified by treatment. Full results from MRI sessions will be reported in a separate study.

## Discussion

To characterize the role of *in utero* glucose availability on fetal heart development in late gestation, the present study employed a mechanistic approach by applying continuous intrafetal glucose infusion to fetuses exposed to maternal undernutrition. Consistent with other models of either early or late‐onset MNR, LGUN resulted in no change to fetal body or heart weight (Burrage et al., 2008, 2009; Darby et al., 2018; Hawkins et al., 2000; Lie et al., 2013; Pereira Susana et al., 2021; Rae et al., 2002) or variable fetal weights (Steinhauser et al., 2021). While fetal plasma glucose concentrations were reduced in LGUN, intrafetal glucose infusion only partially restored plasma glucose concentrations to controls. This may be explained, in part, by increased fetal or placental glucose utilization in the LGUN+G group, which cannot be accounted for by any changes in fetal body weight, which is consistent with other FGR sheep fetuses receiving glucose infusion (Camacho et al., 2022; Rozance et al., 2009). Despite this factor altered fetal glucose availability influences cardiac development at the molecular level by regulating key metabolic, contractility and hormonal factors.

In the cases of human FGR, TH T3 and T4 plasma concentrations are reduced in the last trimester of pregnancy (Kilby et al., 1998), a time during which there should be a developmental surge to promote the exit of CMs from the proliferative growth phase to growth via hypertrophy (Chattergoon et al., 2012). In our study fetal plasma T3 and T4 were not measured; however in previous models of the same MNR severity, plasma T4 is reduced in mid‐gestation and late‐gestation sheep fetuses (Steinhauser et al., 2021; Vonnahme et al., 2003) and persists in the 1‐day‐old lamb (Johnsen et al., 2013). This may be attributed to fetal hypoglycaemia in FGR altering the fetal thyroid gland structure and the peripheral utilization of THs in late gestation (Andrianakis et al., 1990). Although hypoglycaemia due to LGUN did not impact cardiac T3 or T4 concentrations in the present study, like that of theplacental insufficiency (PI)‐FGR fetal sheep heart (Dimasi, Darby, Cho, et al., 2023), cardiac T4 concentrations were increased in LGUN+G. LGUN+G also had a higher mRNA expression of *DIO1* in the present study, a gene responsible for converting T4 to T3 (Maia et al., 2011), and could increase the capacity for T3 production, which may benefit as the prenatal surge of T3 facilitates normal heart maturation (Chattergoon, 2019; Chattergoon et al., 2023, 2012; Drake et al., 2023).

Fetal and maternal glucose infusion mitigates deficits in fetal plasma IGF1 concentrations during maternal starvation (Bassett et al., 1990; Oliver et al., 1993). A limitation of our study is that circulating IGFs were not measured in fetal plasma; however LGUN+G increased cardiac *IGF1* mRNA expression. Interestingly T4 administration also increases plasma and cardiac IGF1 concentrations in fetal pigs (Latimer et al., 1993), and IGF1 infusion increases CM proliferation in fetal sheep (Sundgren et al., 2003). Collectively LGUN+G may promote IGF1‐mediated proliferation of CMs, potentially via T4‐dependent mechanisms. Therefore targeting this mechanism may enhance CM numbers/endowment in FGR fetuses that often have reduced circulating IGF1 concentrations, as reported in human, sheep and guinea‐pig fetuses (Dong & Thompson, 2006; Dwyer & Stickland, 1992; Jonker et al., 2018; Lassarre et al., 1991; Owens et al., 1994), and thus reduce the CVD‐risk profile in FGR‐born adults (Botting et al., 2014; Bubb et al., 2007; Morrison et al., 2007; Vranas et al., 2017; Wang et al., 2011). Consistent with this interpretation increases in pGSK3β in LGUN+G in the present study are also known to induce hyperproliferation of CMs during development (Cheng et al., 2011).

The delayed CM maturational state of FGR fetal hearts often matches their delayed metabolic state with a compromise in the normal developmental switch from utilizing glucose to fatty acids via OXPHOS in late gestation (Chattergoon et al., 2023; Drake et al., 2023). This includes reduced mitochondrial OXPHOS abundance/respiration as reported in many late‐gestation FGR hearts (Chang et al., 2024; Dimasi et al., 2021; Dimasi, Darby, Cho et al., 2023; Pereira Susana et al., 2021; Smith et al., 2022; Song et al., 2021). Consistent with this LGUN decreased the abundance of OXPHOS complex 1 and 3 proteins compared to controls. However in contrast to LGUN OXPHOS complexes 2 and 4 are downregulated in the PI‐FGR heart (Dimasi, Darby, Cho, et al., 2023), highlighting that fetal hypoglycaemia and hypoxaemia may impact the abundance of distinct OXPHOS complexes differently. Indeed LGUN+G restored the abundance of OXPHOS complex 3 to that of controls, which would benefit the aerobic adult heart that primarily utilizes OXPHOS. It is possible that this was achieved via mitochondrial remodelling because increasing fetal glucose supply in the LGUN+G group also increased MFN2 abundance, which promotes outer mitochondrial membrane fusion events and enlargement of the inner mitochondrial cristae space, a factor that corresponds to improved OXPHOS bioenergetics (Pich et al., 2005).

Interestingly selective deficits of OXPHOS complex 3, but not complex 1, are associated with a more rapid progression of diabetic phenotypes (Lang et al., 2023). Thus selective OXPHOS complex 3 restoration in LGUN+G may partially protect the LGUN fetus against cardiac insulin resistance, which often precedes systemic insulin resistance, a phenotype consistent with FGR (McMillen & Robinson, 2005) and observed in MNR‐born baboon juvenile offspring (Choi et al., 2011). Although LGUN reduced insulin‐dependent GLUT4 abundance consistent with changes in the PI‐FGR sheep heart (Dimasi, Darby, Cho, et al., 2023), they persisted in LGUN+G. This could be attributed to intrafetal glucose infusion over a similar gestational period having no impact on cardiac GLUT4 (Darby et al., 2023). Another explanation may be that plasma insulin concentrations are typically reduced in FGR fetuses due to hypoglycaemia and that this may not have been restored in LGUN+G, which is supported by a similar study that infused glucose for 2 weeks into the early‐onset PI‐FGR fetus but was unable to return insulin to control concentrations (Rozance et al., 2009).

One possibility is that the LGUN+G fetal heart preferentially oxidized endogenous glycogen over the infused exogenous glucose, inducing a feedback mechanism that reduced GLUT4 recruitment as it became less reliant on exogenous glucose in LGUN+G (Goodwin et al., 1996; Goodwin et al., 1998). Indeed the PI‐FGR fetal heart has increased glycogen content in late gestation, indicating altered glycogen handling is an adaptive response to hypoglycaemia and/or hypoxaemia (Barry et al., 2006). Here LGUN did not change glycogen content, which may be attributed to the lack of hypoxaemia and differences in the timing and duration between the *in utero* insults. However glycogen content was reduced in LGUN+G, suggesting glycogen was preferentially oxidized over infused exogenous glucose, which is advantageous for the rapid production of ATP while consuming less oxygen than FAs for the same energy converted to mechanical work (Kassiotis et al., 2008). The relationship between T4 and glycogen content only in LGUN+G may indicate an underlying hormonal mechanism with other studies relating cardiac glycogen to plasma T4 in late‐gestation sheep fetuses (Forhead et al., 2009) and T4 administration reducing glycogen content in fetal rabbit heart (Devaskar et al., 1985).

The decreased glycogen accumulation could further improve cardiac function in LGUN+G fetuses, as this may reduce myocardial stiffness (Mellor et al., 2021). The positive relationship between glycogen and fibrosis marker *TIMP1* previously increased in the LGUN fetal RV supports this (Darby et al., 2018) but is not present in the LV in the current study. We also show that the hypertrophy/contractility marker p‐CAMKII increased in the LGUN fetal LV, similar to the RV (Darby et al., 2018). Glucose infusion restoring p‐CAMKII in the LGUN LV to that of control and the negative relationship shared with fetal glucose concentrations highlight a glucose‐dependent role in regulating fetal cardiac p‐CAMKII. The positive correlation between fetal LV cardiac output and p‐CAMKII, a surrogate marker of cardiac function, suggests that the increase in p‐CAMKII in the fetal LV/RV (Darby et al., 2018) is one mechanism that may underly the increased biventricular ejection fraction recently reported in the LGUN fetus (Cho et al. under review). Indeed increased ATP yield would be needed to support this increased ejection fraction, which would be difficult considering OXPHOS abundance is reduced; however it is known that the FGR fetus employs many adaptations to preserve maximal cardiac output levels, termed ‘heart‐sparing’, including a 2–3 fold increase in coronary blood flow (Downey, 1976; Reller et al., 1992a, 1992b).

This study also provides evidence that increases in p‐CAMKII in the PI‐FGR fetal LV (Dimasi, Darby, Cho et al., 2023) may be due to fetal hypoglycaemia rather than hypoxaemia. Nonetheless increased p‐CAMKII persists postnatally in 3‐week‐old sheep offspring born to PI‐FGR (Wang et al., 2015), which may further continue and be a mechanism underlying contractile dysfunction in 3‐month‐old rodent offspring (Harvey et al., 2015) and young adult NHP offspring born to MNR (Kuo, Li, Huber et al., 2017; Kuo, Li, Li, 2017). The lack of change in markers of pathological hypertrophy upstream of p‐CAMKII (e.g. IGF2R) or fibrosis in the LGUN fetal LV in contrast to our previous reports in the LGUN fetal RV (Darby et al., 2018) may be due to increased fetal mean arterial blood pressure (Darby et al., 2018; Edwards & McMillen, 2001) that was not present in the current study, as this is known to contribute to pathological hypertrophy.

There are several limitations of the present study that should be acknowledged. Firstly CMs were not isolated; neither CM size, number nor their nucleation state was determined, which are all factors that could influence the cardiac profile. To this end the predominantly male LGUN+G group should be considered when interpreting the findings, as sexual dimorphism in the expression of molecules that regulate cardiometabolic development has been identified in the FGR heart (Darby et al., 2022; Dimasi, Darby, Cho, et al., 2023; Pereira Susana et al., 2021; Smith et al., 2022; Song et al., 2021). Although the glucose infusion protocol used in this study led to fetal plasma glucose concentrations in LGUN+G being similar to controls, they were also similar to LGUN. Therefore if glucose concentrations had been higher than in LGUN, more of the LGUN‐induced alterations may have been corrected. This may require monitoring fetal glucose concentrations in real time, adjusting the glucose infusion to maintain euglycaemia (Camacho et al., 2022; Rozance et al., 2009) or increasing the overall glucose infusion rate. However it is also possible that the placental or fetal tissues would take up the infused exogenous glucose to promote growth, thereby inhibiting a normalization of plasma glucose concentrations. Finally this study was focused on the mechanistic role of glucose in the setting of LGUN; thus the reduced delivery of other substrates (e.g. amino and fatty acids, insulin, lactate, ketones) induced by LGUN, but not restored by glucose infusion, may still play a pivotal role in the impact of maternal undernutrition on fetal cardiac development.

## Conclusion

This study highlights the importance of separating the roles of oxygen and glucose in understanding the regulation of fetal heart development. Specifically it revealed the role of glucose availability in regulating fetal hormonal, metabolic and contractility profiles such that reduced fetal glucose concentrations during MNR are a key mechanism contributing to molecular signatures that programme a CVD‐risk profile. Notably the poor cardiac profile in LGUN occurred without any change in fetal body or heart weights, indicating that changes in these factors alone cannot govern the CVD‐risk profile. Our findings reaffirm the importance of adequate maternal nutrition in late gestation to ensure normal fetal heart development and reduce the risk of CVD across the life course.

## Additional information

## Competing interests

The authors have no competing interests to report.

## Author contributions

Conception or design of the work: J.R.T.D., S.L.H., C.K.M., M.S., J.L.M. Acquisition or analysis or interpretation of data for the work: all authors. Drafting the work or revising it critically for important intellectual content: all authors. Final approval of the version to be published and agreement to be accountable for all aspects of the work: all authors.

## Funding

Janna L. Morrison and the molecular work were funded by an Australian Research Council Future Fellowship (level 3, FT170100431). MRB is funded by an Australian Research Training Program Scholarship.

## Supporting information


Peer Review History

## Data Availability

Data supporting the findings of this paper are available from the authors upon reasonable request.
