# Supplementary material for: Fetal glucose availability: a key regulator of the metabolic, hormonal and contractility profiles of the fetal sheep heart
Source: J Physiol. 2025 May 28;604(16):6817–39. doi: 10.1113/JP288303 (PMC13481007; doi:10.1113/JP288303)
Supplement: Supplementary file 1 [file TJP-604-6817-s001.pdf]

The Journal of Physiology

Fetal glucose availability: A key regulator of the metabolic, hormonal and contractility profile of the fetal sheep heart

Melanie Rose Bertossa, Jack RT Darby, Stacey L Holman, Steven KS Cho, Ashley S Meakin, Mitchell C Lock, Jordan Arthur Minns, Michael D Wiese, Christopher K Macgowan, Mike Seed, and Janna L Morrison
DOI: 10.1113/JP288303

Corresponding author(s): Janna Morrison (janna.morrison@unisa.edu.au)

The referees have opted to remain anonymous.

Review Timeline:

Submission Date:	14-Jan-2025
Editorial Decision:	27-Feb-2025
Revision Received:	04-Apr-2025
Accepted:	29-Apr-2025

Senior Editor: Bjorn Knollmann

Reviewing Editor: Christopher Lear

Transaction Report:

(Note: With the exception of the correction of typographical or spelling errors that could be a source of ambiguity, letters and reports are not edited. Depending on transfer agreements, referee reports obtained elsewhere may or may not be included in this compilation. Referee reports are anonymous unless the Referee chooses to sign their reports.)

Dear Janna,

Re: JP-RP-2025-288303 "Fetal glucose availability: A key regulator of the metabolic, hormonal and contractility profile of the fetal sheep heart" by Melanie Rose Bertossa, Jack RT Darby, Stacey L Holman, Steven KS Cho, Ashley S Meakin, Mitchell C Lock, Jordan Arthur Minns, Michael D Wiese, Christopher K Macgowan, Mike Seed, and Janna L Morrison

Thank you for submitting your manuscript to The Journal of Physiology. It has been assessed by a Reviewing Editor and by 2 expert referees and we are pleased to tell you that it is acceptable for publication following satisfactory revision.

The referee reports are copied at the end of this email.

Please address all the points raised and incorporate all requested revisions or explain in your Response to Referees why a change has not been made. We hope you will find the comments helpful and that you will be able to return your revised manuscript within 4 weeks. If you require longer than this, please contact journal staff: jp@physoc.org.

Your revised manuscript should be submitted online using the link in your Author Tasks Link Not Available. This link is accessible via your account as Corresponding Author; it is not available to your co-authors. If this presents a problem, please contact journal staff (jp@physoc.org). Image files from the previous version are retained on the system. Please ensure you replace or remove any files that are being revised.

If you do not wish to submit a revised version of your manuscript, you must inform our journal staff (jp@physoc.org) or reply to this email to request withdrawal. Please note that a manuscript must be formally withdrawn from the peer review process at one journal before it may be submitted to another journal.

TRANSPARENT PEER REVIEW POLICY: To improve the transparency of its peer review process The Journal of Physiology publishes online, as supporting information, the peer review history of all articles accepted for publication. Readers will have access to decision letters, including Editors' comments and referee reports, for each version of the manuscript, as well as any author responses to peer review comments. Referees can decide whether or not they wish to be named on the peer review history document.

ABSTRACT FIGURES: Authors are expected to use The Journal's premium BioRender account to create/redraw their Abstract Figures. Information on how to access this account is here:
https://physoc.onlinelibrary.wiley.com/journal/14697793/biorender-access.

This will enable Authors to create and download high-resolution figures. If authors have used the free BioRender service, they can use the instructions provided in the link above to download a high-resolution version suitable for publication.
The link provided should only be used for the purposes of this submission. Authors will be charged for figures created on this account if they are not related to this manuscript submission.

LANGUAGE EDITING AND SUPPORT FOR PUBLICATION: If you would like help with English language editing, or other article preparation support, Wiley Editing Services offers expert help, including English Language Editing, as well as translation, manuscript formatting, and figure formatting at www.wileyauthors.com/eeo/preparation. You can also find resources for Preparing Your Article for general guidance about writing and preparing your manuscript at www.wileyauthors.com/eeo/prepresources.

RIGOUR AND REPRODUCIBILITY: We recommend authors consult The Journal's Rigour and Reproducibility requirements to ensure that their manuscript is suitable for publication: https://physoc.onlinelibrary.wiley.com/pb-assets/hub-assets/physoc/documents/TJP-Rigour-and-Reproducibility-Requirements.pdf

REVISION CHECKLIST:
Check that your Methods section conforms to journal policy: https://jp.msubmit.net/cgi-bin/main.plex?form_type=display_requirements#methods

Check that data presented conforms to the statistics policy: https://jp.msubmit.net/cgi-bin/main.plex?form_type=display_requirements#statistics

Upload a full Response to Referees file. To create your 'Response to Referees' copy all the reports, including any comments from the Senior and Reviewing Editors, into a Microsoft Word, or similar, file and respond to each point, using font or background colour to distinguish comments and responses and upload as the required file type.

Please upload two versions of your manuscript text: one with all relevant changes highlighted and one clean version with no changes tracked. The manuscript file should include all tables and figure legends, but each figure/graph should be uploaded as separate, high-resolution files. The journal is now integrated with Wiley's Image Checking service. For further details, see: https://www.wiley.com/en-us/network/publishing/research-publishing/trending-stories/upholding-image-integrity-wileys-image-screening-service

You may also upload:
- 'Potential Cover Art' for consideration as the issue's cover image
- Appropriate Supporting Information (Video, audio or data set: see https://jp.msubmit.net/cgi-bin/main.plex?form_type=display_requirements#supp).

We look forward to receiving your revised submission.

If you have any queries, please reply to this email and we will be pleased to advise.

Best wishes,

Bjorn Knollmann
Senior Editor
The Journal of Physiology

REQUIRED ITEMS

- Author photo and profile. First or joint first authors are asked to provide a short biography (no more than 100 words for one author or 150 words in total for joint first authors) and a portrait photograph. These should be uploaded and clearly labelled together in a Word document with the revised version of the manuscript. See Information for Authors for further details.

- Please upload separate high-quality figure files via the submission form.

- You must upload original, uncropped western blot/gel images (including controls) if they are not included in the manuscript. This is to confirm that no inappropriate, unethical or misleading image manipulation has occurred. These should be uploaded as 'Supporting information for review process only'. Please label/highlight the original gels so that we can clearly see which sections/lanes have been used in the manuscript figures. For more information, see: https://physoc.onlinelibrary.wiley.com/hub/journal-policies#imagmanip.

- Please include an Abstract Figure file, as well as the Figure Legend text within the main article file. The Abstract Figure is a piece of artwork designed to give readers an immediate understanding of the research and should summarise the main conclusions. If possible, the image should be easily 'readable' from left to right or top to bottom. It should show the physiological relevance of the manuscript so readers can assess the importance and content of its findings. Abstract Figures should not merely recapitulate other figures in the manuscript. Please try to keep the diagram as simple as possible and without superfluous information that may distract from the main conclusion(s). Abstract Figures must be provided by authors no later than the revised manuscript stage and should be uploaded as a separate file during online submission labelled as File Type 'Abstract Figure'. Please also ensure that you include the figure legend in the main article file. All Abstract Figures should be created using BioRender. Authors should use The Journal's premium BioRender account to export high-resolution images. Details on how to use and access the premium account are included as part of this email.

EDITOR COMMENTS

Reviewing Editor:

Thank you for your submission to The Journal. The manuscript was generally well received by our reviewers, please see their comments on limitations and areas that need improvement.

Further to these points

-Please improve your key point summary to include background information and relevance rather than starting with results

-Please make it more clear that MRI was performed (the provided methodology is important for understanding any confounding) but the results are not presented here.

-As the reviewers have both requested, please more clearly acknowledge that your glucose infusion did not normalise glucose levels, an explanation for this, and the potential implications when interpreting the present findings. Would the

authors be intending to repeat this study with a protocol that truly normalises glucose?

Please also see 'Required Items' above.

Senior Editor:

I concur with the reviewing editor. Please address each of the reviewers concerns and revise the manuscript accordingly.

REFEREE COMMENTS

Referee #1:

Overall, this is a very well performed study and the manuscript well written. The manuscript attempts to better understand the role of glucose as opposed to hypoxia in regulating fetal outcomes in animals impacted by nutrient restriction. The infusion of glucose to the fetus is only possible in a model such as this and is a real strength of the study which offsets any limitations such as lower sample size given that any meaningful effects would be quite large. I was surprised that the effectiveness of the glucose infusion on modifying fetal glucose was relatively low but this can be explained by changes to homeostatic mechanisms. I do have a number of comments for the authors to address

1. Why would LGUN increase brain weight without impacting body weight? Where have the reallocated nutrients come from? Is the mother largely buffering the calorie deficit (except for fetal glucose) and is the brain weight increase driven by endocrine factors attempting to preserve brain size rather than nutrient or oxygen reallocation. For example, low glucose drives increased insulin which impacts tissue proliferation. I also note you measured IGF2 mRNA in heart tissue and this was different. Could changes in circulating IGF2 explain the brain sparing?

2. Given the main tissue changes notes were changes to brain size, I was surprised that the study did not further investigate brain tissues to try and see what was happening.

3. I note you have indicated I the methods that fasting blood glucose before diet allocation was taken and then in the table you report maternal mean blood glucose. Can you confirm the timing of this glucose measurement? What was the impact of the LGUN on maternal glucose and insulin taken at the end of the protocol? I am just trying to gauge if the effect on fetal glucose was due to a drop in maternal glucose/hormones or placental transport secondary to lower maternal nutrition/body composition.

4. Although not significant, why were slightly larger animals used for the LGUN and LGUN +G groups?

5. Throughout the paper, most of the measurements are fetal. Are you able to make this clear in table and figure captions (ie table 2, I assume this is fetal based on methods and the fact that the text related to this is in fetal outcomes but make the table stand alone). Similarly, make sure you highlight the tissue when doing qPCR data or western blot data (again I know it's the heart but add it into he table and figure information).

6. The fetal glucose infusion had minimal impacts on fetal blood glucose and while it is no longer statistically different from control it is much closer to the LGUN than it is to control. Could you have used a higher glucose infusion? Has the setpoint of glucose homeostasis shifted lower by LGUN and LGUN=G cannot really increase this? You comment early in the discussion that this may be increased placental glucose utilisation or usage by other tissues. Was this able to be measured in anyway? Maybe even things like weights of those tissues, body composition etc

7. The endocrine changes in the heart tissue are really interesting. Could the increase in T4 just be a failure of deiodination to T3 and reduced thyroid hormone signalling? I note you say in the discussion that DIO1 was reportedly higher in a previous paper of this model which goes against the accumulation of T4 without it changing to the function hormone T3.

8. What could the progesterone changes mean? Have you previously shown changes to circulating progesterone? Could these tissue levels are reflective of those? If so what might changes to circulating progesterone indicate? Perhaps a change in perceived gestational age as progesterone changes over pregnancy (accelerated or slowed fetal development relative to placental function). Could the progesterone be regulating anything in the fetal heart?

9. The mitochondrial complex data is really interesting and convincing. It would have been fantastic to assess oxygen flux through the chain when inhibiting different complexes (Seahorse etc) but given the amount of other data, I feel this is sufficient.

Referee #2:

The objective of the current study is to investigate the role of glucose availability in fetal heart development. This is a well written manuscript, but I have a few suggestions below for clarity. A main limitation in the experimental design was that the intra-glucose infusion did not increase fetal glucose concentrations significantly compared to the LGUN group. This is somewhat surprising because of the direct infusion into the fetus and ability to monitor glucose concentrations closely. Can you explain your target glucose values? Or how the glucose infusion rate was chosen? Also the changes in the maternal mean glucose concentrations were not different, which raises the point on whether undernutrition was the best model for investigating glucose availability for heart development. I understand that the fetal sex was not analyzed do to then low #f in the +G group. However, I cannot help to note that the complex 3 levels in the male fetuses are greater and may explain the difference the LGUN+G fetal hearts. It might be wise to look at males only to validate this point.

Suggested edits:

Line 142 spelling output

Line 160 the surgery days listed don't coincide with the group assignments at 110-111 dGA. Please explain

Line 184 if fetal sex was considered in the assignment randomization, then why is the LGUN+G group mismatched-only 1 female. The power analysis for the groups should be included to meet the ARRIVE guidelines.

Line 222 and 235 and 301 how were the subsets chosen? Describe method for unbiased selection.

Line 405 for mean glucose concentrations can you specify days (pre and post LGUN or only after treatment started)

Table 1 it is curious that the Hb and Hct don't correlate with one another better

---

END OF COMMENTS

EDITOR COMMENTS

Reviewing Editor:

Thank you for your submission to The Journal. The manuscript was generally well received by our reviewers, please see their comments on limitations and areas that need improvement.

Further to these points

-Please improve your key point summary to include background information and relevance rather than starting with results

We thank the editor for raising this point. The key point summary has been edited to include an additional first point.

- **Line 38-42:** "Maternal late gestation undernutrition (LGUN) reduces fetal plasma glucose concentrations. To investigate the role of glucose availability in fetal left ventricle (LV) development, we assessed whether LGUN-induced alterations in the contractility, metabolic, and hormonal profile can be ameliorated in LGUN fetuses receiving glucose infusion (LGUN+G).

-Please make it more clear that MRI was performed (the provided methodology is important for understanding any confounding) but the results are not presented here.

Thank you for raising this. We hope that the addition of the above first key point summary highlights the focus of this study, which was to investigate the impact of LGUN and LGUN+G on the signalling pathways within the left ventricle governing cardiac development using a range of molecular techniques. Due to the large size of the two datasets, the full MRI results will be published in a separate and comprehensive manuscript, which will include fetal haemodynamics, oxygen delivery, and cardiac function. This is keeping with the principal of the 3 Rs in that multiple scientific aims are being addressed using this experimental protocol without increasing the total number of animals involved (Grundy, 2015). Moreover, given that MRI-acquired data on fetal cardiac function were available, we had the exciting opportunity to correlate these functional measures with the expression and abundance of key signaling molecules within the fetal left ventricular tissue itself (shown in Figure 6C). For clarity, additional text has been added to the end of the methods section, detailing the MRI, and the results section that utilised the MRI data.

Line 260: "Each MRI session was part of an extensive data collection protocol, and full MRI results will be reported in a separate study. In this study, the fetal cardiac function outcomes are correlated with cardiac molecular findings."

Line 532: "Full results from MRI sessions will be reported in a separate study."

-As the reviewers have both requested, please more clearly acknowledge that your glucose infusion did not normalise glucose levels, an explanation for this, and the potential implications when interpreting the present findings. Would the authors be intending to repeat this study with a protocol that truly normalises glucose?

We thank the senior editors and reviewers for raising this issue and acknowledge that glucose infusion did not restore fetal glucose concentration in LGUN. We agree that a more explicit acknowledgment of the lack of normalisation should be added throughout the manuscript.

The second key point summary addresses this by adding the following text:

Line 44-45: "despite fetal glucose infusion only partially normalising fetal plasma glucose concentrations to that of Controls"

Paragraph 1 of the discussion acknowledges this lack of fetal plasma glucose concentration normalisation by intrafetal glucose infusion with the following text:

Line 544-542: "intrafetal glucose infusion only partially restored plasma glucose concentrations compared to Controls. This may be explained, in part, by increased fetal or placental glucose utilisation in the LGUN+G group"

Furthermore, re-acknowledgment of this and suggestions for future alterations to the existing glucose infusion protocol have been added to the limitation paragraph of the discussion with the following text:

Line 661-664: "While the glucose infusion protocol used in this study led to fetal plasma glucose concentrations in LGUN+G being similar to Controls, they were also similar to LGUN. Therefore, if glucose concentrations had been higher than in LGUN, more of the LGUN-induced alterations may have been corrected. This may require monitoring fetal glucose concentrations in real-time, adjusting the glucose infusion to maintain euglycemia (Rozance *et al.*, 2009; Camacho *et al.*, 2022), or increasing the overall glucose infusion rate. However, it is also possible that the placental or fetal tissues would take up the infused exogenous glucose to promote growth, thereby inhibiting a normalisation of plasma glucose concentrations."

One mechanism explaining this lack of normalisation using the present infusion protocol could be the placental transfer of the "exogenous" glucose from the fetal to the maternal side. Given that placental glucose transfer occurs primarily via concentration gradient and that maternal glucose concentrations are higher than fetal concentrations, this theory remains unlikely. We believe this lack of normalisation can be explained by the heterogeneity in which each LGUN+G fetus utilised the exogenous glucose. Moreover, the impact of constitutionally larger/smaller fetuses on this study cannot be ruled out. The variability in the way glucose was used may be attributable to each fetus' genetically predefined growth potential, with those fetuses predestined to have higher growth potential increasing their glucose uptake more than those with a slightly lower growth potential. Moreover, while there

was likely a degree of variability in glucose utilisation, the normalisation of brain to body weight ratio and the expression of some cardiac proteins in the LGUN+G group indicate some of the infused glucose was used for fetal growth.

Please also see 'Required Items' above.

Senior Editor:

I concur with the reviewing editor. Please address each of the reviewers concerns and revise the manuscript accordingly.

REFEREE COMMENTS

Referee #1:
Overall, this is a very well performed study and the manuscript well written. The manuscript attempts to better understand the role of glucose as opposed to hypoxia in regulating fetal outcomes in animals impacted by nutrient restriction. The infusion of glucose to the fetus is only possible in a model such as this and is a real strength of the study which offsets any limitations such as lower sample size given that any meaningful effects would be quite large. I was surprised that the effectiveness of the glucose infusion on modifying fetal glucose was relatively low but this can be explained by changes to homeostatic mechanisms. I do have a number of comments for the authors to address

1. Why would LGUN increase brain weight without impacting body weight? Where have the reallocated nutrients come from? Is the mother largely buffering the calorie deficit (except for fetal glucose) and is the brain weight increase driven by endocrine factors attempting to preserve brain size rather than nutrient or oxygen reallocation. For example, low glucose drives increased insulin which impacts tissue proliferation. I also note you measured IGF2 mRNA in heart tissue and this was different. Could changes in circulating IGF2 explain the brain sparing?
We thank the reviewer for raising this point. The increase in brain weight is a particularly interesting outcome of the study, and we have given it considerable thought; however, it may not be possible for us to address the issue entirely. This study was born from the results of a previous study that demonstrated LGUN impacts on cardiac growth (Darby *et al.*, 2018). This led us to wonder if the underlying factor of maternal global undernutrition in sheep was a decrease in fetal plasma glucose. Thus, we infused glucose at a level we had previously shown to impact fetal growth and plasma concentrations. The increase in relative brain weight was negatively correlated with fetal plasma glucose concentrations but only in LGUN (Figure 1F). Thus, while glucose concentrations are low in the LGUN fetus, there is a compensatory hemodynamic response to redistribute blood flow and substrates, such as

glucose, toward the brain, prioritising growth. This circulatory redistribution is characteristic of the fetal brain-sparing response that has been shown previously in other models of maternal nutrient restriction (Williams et al., 2005; Wilson et al., 2021) and is a primary focus of a separate manuscript focused on MRI outcomes. A limitation of this study is that hormones such as IGF-1/2 in circulation were not measured; however, we did measure these important growth factors and their receptors in the heart tissue itself. The following text was added to the discussion to highlight this as a limitation of the study:

Line 571-573: "A limitation of our study is that circulating IGFs were not measured in fetal plasma; however, LGUN+G increased cardiac *IGF1* mRNA expression*."*

2. Given the main tissue changes notes were changes to brain size, I was surprised that the study did not further investigate brain tissues to try and see what was happening.

We agree that further analysis of the brain tissue would provide insight into the physiological interactions between the heart and brain (i.e., hormonal and haemodynamic mechanisms) and a better overall understanding of the fetal response to LGUN. However, the primary focus of the present study was the fetal heart. The fetal brains from this cohort of animals are currently the focus of a forthcoming manuscript, including a large dataset of molecular outcomes, allowing these mechanisms to be interlinked in the future.

3. I note you have indicated in the methods that fasting blood glucose before diet allocation was taken and then in the table you report maternal mean blood glucose. Can you confirm the timing of this glucose measurement? What was the impact of the LGUN on maternal glucose and insulin taken at the end of the protocol? I am just trying to gauge if the effect on fetal glucose was due to a drop in maternal glucose/hormones or placental transport secondary to lower maternal nutrition/body composition.

We thank the reviewer for raising this. The maternal glucose measurement reported in the table is mean gestational blood glucose (i.e., the average of blood glucose concentrations obtained from daily blood sampling throughout the experimental protocol). For clarity, the word "gestational" has been added to Table 1. In addition, we acknowledge that it may have been difficult to locate this information regarding maternal sampling as the previous method title referred to fetal blood sampling only. To highlight this, the title of the methods section detailing maternal sampling has been changed.

Line 210: "Maternal and fetal blood collection".

In a subset of animals, we measured maternal insulin at ~134dGA (See figure 1 below), of which the protocol endpoint was ~139-142dGA. The data shows that LGUN did not decrease maternal insulin compared to controls but that fetal glucose infusion in LGUN ewes did decrease maternal insulin concentration (Figure 1). Thus, approximately 20 days into the experimental protocol, LGUN+G, but not LGUN, decreased plasma insulin in the ewe. While we acknowledge that glucose infusion decreased insulin concentrations, we have previously shown maternal cardiovascular adaptations in response to LGUN redirect blood flow from

the periphery to the uteroplacental unit, preserving placental growth/function and concomitantly increasing the delivery of maternal substrates to the fetus (Meakin *et al.*, 2022; Cho *et al.*, 2024). It should also be noted that maternal insulin concentrations were not different between the LGUN and LGUN+G groups. Moreover, maternal mean gestational blood glucose was not statistically different between the treatment groups.

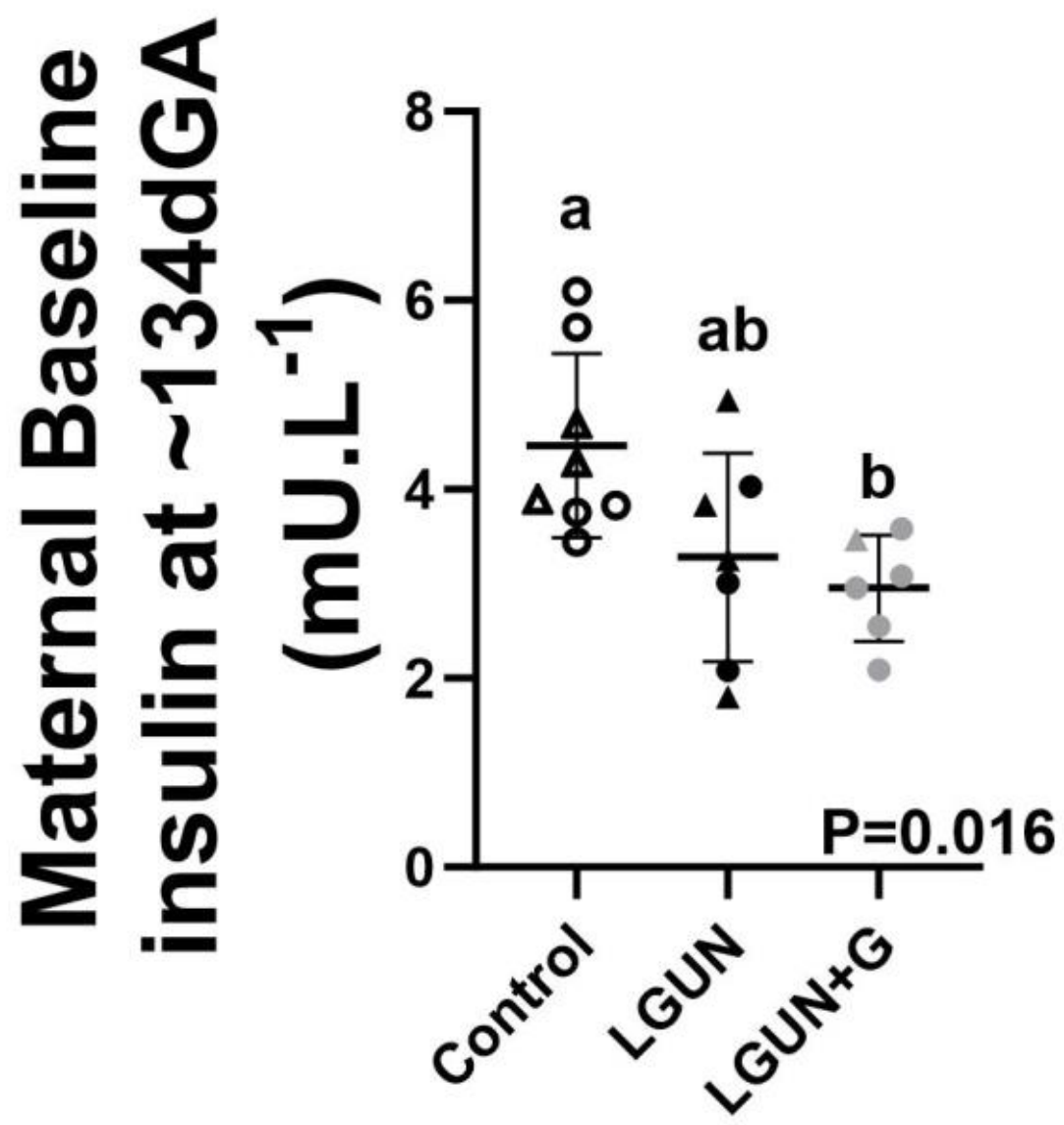

Figure 1: Maternal baseline insulin measured at ~134dGA
Data expressed as mean ±SD and analysed by One-way ANOVA followed by a *post hoc* Bonferroni's multiple comparisons test. ns, *P>0.05*. Superscript alphabetical letters indicate significant differences between treatment groups (*P<0.05*), such that values with different alphabetical letters are statistically different, and those with the same alphabetical letters are not different. Circles=males (m); triangles=females (f). Controls in open data points (protein n=8, 6m, 3f), LGUN in black data points (n=8, 4m, 4f), and LGUN+G in grey data points (n=6, 5m, 1f).

4. Although not significant, why were slightly larger animals used for the LGUN and LGUN +G groups?
Ewes were randomly assigned by minimisation based on maternal weight, maternal fasting blood glucose, and fetal sex to one of three groups. However, not all fetuses survived to the end of the protocol (Cho *et al.*, 2024). Thus, there is some variation in maternal weight at the time of allocation.

5. Throughout the paper, most of the measurements are fetal. Are you able to make this clear in table and figure captions (ie table 2, I assume this is fetal based on methods and the fact that the text related to this is in fetal outcomes but make the table stand alone). Similarly, make sure you highlight the tissue when doing qPCR data or western blot data (again I know it's the heart but add it into the table and figure information).

We thank the reviewer for bringing this to our attention. To improve clarity, the title for Table 2 has been amended to include the word ‘fetal.’

Line 436: Table 2. The impact of LGUN and LGUN+G on basal **fetal** blood pressure between 118-119d and 137-138dGA**.**

To highlight the tissue type from which the results are obtained throughout the manuscript, “in the fetal LV” or “cardiac” has been added to all relevant results and figure legends.

6. The fetal glucose infusion had minimal impacts on fetal blood glucose and while it is no longer statistically different from control it is much closer to the LGUN than it is to control. Could you have used a higher glucose infusion? Has the setpoint of glucose homeostasis shifted lower by LGUN and LGUN+G cannot really increase this? You comment early in the discussion that this may be increased placental glucose utilisation or usage by other tissues. Was this able to be measured in anyway? Maybe even things like weights of those tissues, body composition etc

We thank the reviewer for raising this and hope that the many revisions made to the manuscript based on similar feedback from the senior editor improve its quality. This includes the acknowledgment of the lack of normalisation of glucose throughout the manuscript and additional text added to the discussion that suggests future amendments that could be made to the existing protocol, such as monitoring fetal glucose concentrations in real-time and a higher glucose infusion.

We had previously performed a study where we infused glucose into the fetal circulation in normal pregnancy. In this study, there was an effect on lung development and appetite regulation by the brain but very few effects on the fetal heart (Mühlhäusler *et al.*, 2005; McGillick *et al.*, 2014; Darby *et al.*, 2023). In that study, glucose was infused into the fetus for 10 days, resulting in a doubling of fetal glucose concentration from 1.1 mmol/L in controls to 2.2 mmol/L in glucose-infused fetuses. Thus, it is interesting that a similar glucose infusion in this study did not increase fetal glucose concentration in undernourished ewes.

The increase in glucose utilisation by the placenta was only speculated upon, as glucose was not isotopically labelled, and fetal umbilical vessels were not catheterised, which limited our ability to estimate the amount of glucose moving across the placenta. Although outside the scope of the present study, we aim to conduct additional analyses on these tissues to quantify glucose transport expression and glycogen content, which will provide further insight into the present study.

7. The endocrine changes in the heart tissue are really interesting. Could the increase in T4 just be a failure of deiodination to T3 and reduced thyroid hormone signalling? I note you say in the discussion that DIO1 was reportedly higher in a previous paper of this model which goes against the accumulation of T4 without it changing to the function hormone T3.

In this study, we report an increase in *DIO1*. We have clarified the following:

Line 563: LGUN+G also had higher mRNA expression of *DIO1* in the present study.

We acknowledge that the increase in cardiac T4 concentrations could be a failure of deiodination to T3 and that an increase in cardiac DIO1 expression could go against the accumulation of cardiac T4, as suggested by the reviewer. However, it is also likely that given the evidence that hypoglycaemia induced by MNR reduces circulating T4 rather than T3 in fetuses (Vonnahme *et al.*, 2003; Steinhauser *et al.*, 2021), glucose infusion may increase the production of T4 from the fetal thyroid gland, which, in part, explains the accumulation of cardiac T4. It could be speculated that an increase in DIO1 may then lead to an increase in the capacity of this accumulated cardiac T4 to be deiodinated into T3.

8. What could the progesterone changes mean? Have you previously shown changes to circulating progesterone? Could these tissue levels are reflective of those? If so what might changes to circulating progesterone indicate? Perhaps a change in perceived gestational age as progesterone changes over pregnancy (accelerated or slowed fetal development relative to placental function). Could the progesterone be regulating anything in the fetal heart?

Work from our research group has not previously shown changes in circulating fetal progesterone; however, others have shown that FGR fetuses, induced by either placental insufficiency or maternal undernutrition is associated with low progesterone concentrations in both maternal and fetal circulation (Regnault *et al.*, 1999; Bloomfield *et al.*, 2003; Bloomfield *et al.*, 2004; Lekatz *et al.*, 2010; Long *et al.*, 2010; Roman *et al.*, 2013). Therefore, increased cardiac progesterone levels following glucose infusion may reflect an increase in circulating progesterone levels. If progesterone concentrations are increased in the circulation, it may indicate a more stable pregnancy and enhanced placental function, including the mobilisation of glucose and increased placental blood flow. To the best of our knowledge, there is no information on how maternal undernutrition alters progesterone concentrations within the fetal heart tissue itself. Low progesterone concentrations within the heart tissue may alter the capacity for glucose uptake and glycogen storage in the fetal heart, as is observed in endometrial tissue (Zhang *et al.*, 2020).

9. The mitochondrial complex data is really interesting and convincing. It would have been fantastic to assess oxygen flux through the chain when inhibiting different complexes (Seahorse etc) but given the amount of other data, I feel this is sufficient.
Thank you for acknowledging that the current mitochondrial data presented is sufficient. We agree that high-resolution respirometry such as Seahorse or Oroboros experiments to assess mitochondrial function would be a great addition to the study. However, these experiments require fresh tissue (as freeze thaw cycles disrupts the mitochondrial membrane) and we did not have access to a Seahorse or Oroboros at the time the study was performed at the farm.

Further investigation of mitochondrial function using these techniques will be performed in future studies.

Referee #2:

The objective of the current study is to investigate the role of glucose availability in fetal heart development. This is a well written manuscript, but I have a few suggestions below for clarity. A main limitation in the experimental design was that the intra-glucose infusion did not increase fetal glucose concentrations significantly compared to the LGUN group. This is somewhat surprising because of the direct infusion into the fetus and ability to monitor glucose concentrations closely. Can you explain your target glucose values? Or how the glucose infusion rate was chosen? Also the changes in the maternal mean glucose concentrations were not different, which raises the point on whether undernutrition was the best model for investigating glucose availability for heart development. I understand that the fetal sex was not analyzed do to then low #f in the +G group. However, I cannot help to note that the complex 3 levels in the male fetuses are greater and may explain the difference the LGUN+G fetal hearts. It might be wise to look at males only to validate this point.

We thank the reviewer for raising these points. Firstly, the glucose infusion rate was set to target glucose concentrations within the control range and the rate based on previous studies (McGillick *et al.*, 2014; Darby *et al.*, 2023). As described in the above responses to both the senior editor and reviewer 1, the manuscript has been updated to provide further explanation for this lack of normalisation and additional text for future protocol amendments.

It was not possible for us to adjust the glucose infusion to a specific target glucose level as we were limited in our ability to measure glucose levels below 1 mmol/L in real-time at the farm. We use a Siemens blood gas analyser and can measure maternal glucose concentrations, but most fetal glucose concentrations (control and all LGUN) were below the limit of detection. We did consider purchasing a YSI glucose monitor, but the cost was beyond our capacity. For this reason, fetal glucose concentrations were measured upon completion of the study. Even if we had performed this assay as blocks of animals were completed, we would still not have been able to titrate the glucose infusion within a specific animal. Thus, we chose to use a set glucose infusion based on our previous studies in normal pregnancies.

We acknowledge that maternal mean glucose concentrations were not different between groups. However, maternal undernutrition resulted in fetal hypoglycaemia in LGUN ewes in the present study. Moreover, utilising the undernutrition model allowed us to understand the contributions of hypoglycaemia on fetal heart development independent of hypoxemia. This contrasts with other models that can induce fetal growth restriction, such as placental insufficiency, which not only results in hypoglycaemia but also hypoxemia, making it difficult to tease apart the individual roles of different substrates for fetal growth on cardiac development.

We agree that the male-dominant LGUN+G group is a limitation as it is well characterised that mitochondrial phenotypes are sexually dimorphic in FGR fetal hearts (Al-Hasan *et al.*,

2013; Pereira *et al.*, 2021; Song *et al.*, 2021; Darby *et al.*, 2022; Smith *et al.*, 2022; Dimasi *et al.*, 2024). Additionally, we can confirm the reviewer's observation that the same statistical change between treatment groups remains when females are excluded from the complex 3 data (See figure 2 below).

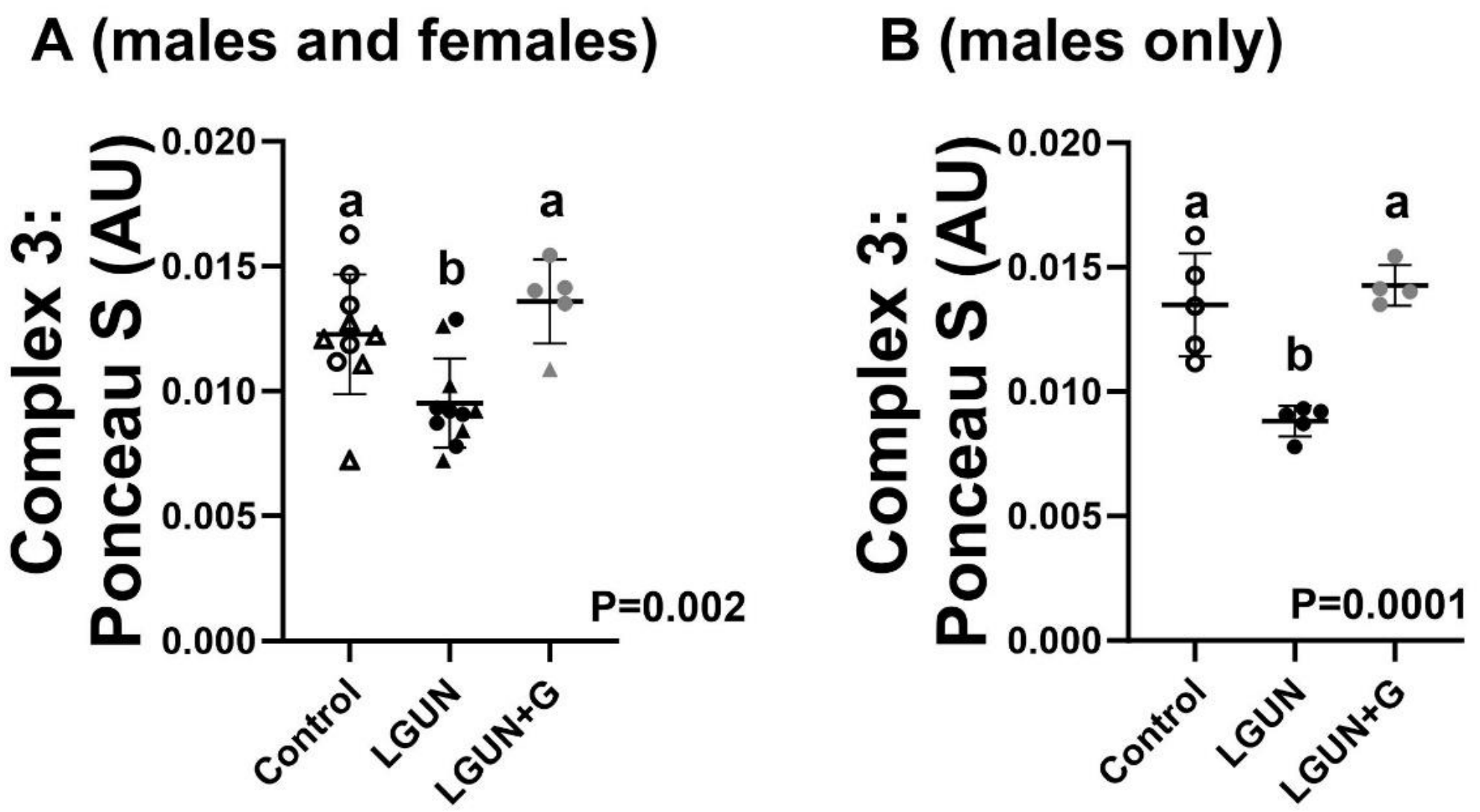

Figure 2: The impact of LGUN and LGUN +G on cardiac oxidative phosphorylation complex 3 in males and females (A) and males only (B).
Data presented as normalised protein expression in arbitrary units (AU), expressed as mean ±SD and analysed by One-way ANOVA followed by a *post hoc* Bonferroni's multiple comparisons test. ns, $P>0.05$. Superscript alphabetical letters indicate significant differences between treatment groups ($P<0.05$), such that values with different alphabetical letters are statistically different, and those with the same alphabetical letters are not different. Circles=males (m); triangles=females (f). Controls in open data points (n=10,5m, 5f), LGUN in black data points (n=11,6m, 5f), and LGUN+G in grey data points (n=6,5m, 1f).

The limitation paragraph has been supplemented with additional text/references to raise further caution for readers interpreting the present study's cardiometabolic data.

Line 655-659: " To this end, the predominantly male LGUN+G group should be considered when interpreting the findings, as sexual dimorphism in the expression of molecules that regulate cardiometabolic development have been identified in the FGR heart (Pereira *et al.*, 2021; Song *et al.*, 2021; Darby *et al.*, 2022; Smith *et al.*, 2022; Dimasi *et al.*, 2023)"

Suggested edits:

Line 142 spelling output

We thank the reviewer for bringing this typo to our attention. The word output has been corrected on line 142.

Line 160 the surgery days listed don't coincide with the group assignments at 110-111 dGA. Please explain

We thank the reviewer for pointing this out. As described, surgery was performed between a range of gestational days (~109-124 days); however, irrespective of the surgery date, all ewes were allocated to their treatment group at 110-111dGA.

Line 184 if fetal sex was considered in the assignment randomization, then why is the LGUN+G group mismatched-only 1 female. The power analysis for the groups should be included to meet the ARRIVE guidelines.

Randomisation by minimisation was based on maternal weight, fasting glucose and, lastly, fetal sex. Due to the high cost of running large, logistically challenging studies in the chronically catheterised pregnant sheep with multiple physiological assessments including MRI, it was not our intention to power this study to determine the impact of fetal sex or the interaction between treatment group and sex. Instead, we attempted to allocate equal numbers of males and females to each group. The original allocation of animals to the LGUN+G group included more females. However, due to the fetal demise of several females in the LGUN+G group between the postoperative period and postmortem (percent loss similar between each treatment group), there are fewer females in this group. Moreover, for transparency, we have indicated the sex of each fetus within each group by using different symbols in graphs (circles, triangles) so that the reader can see for themselves the distribution of males and females from a purely qualitative perspective.

Line 222 and 235 and 301 how were the subsets chosen? Describe method for unbiased selection.

Re Line 222: All animals were initially allocated to undergo fetal blood pressure studies; however, we were unable to measure fetal blood pressure in some animals due to the catheter not being patent or having poor pulsatility. The specific numbers related to exclusion in each treatment group are described in the legend of Table 2:

Line 441: "Exclusion criteria for blood pressure study included catheter not patent at 118dGA (Control n=2) or 138dGA (Control n=3; LGUN n=1; LGUN+G n=1), poor pulsatility at 118dGA (Control n=2; LGUN n=3) or 138dGA (Control n=3; LGUN n=3) and blood pressure study not performed at 118dGA (Control n=2; LGUN n=3) or 138dGA (Control n=3; LGUN n=3)."

Re Line 235: fetuses allocated to MRI were excluded if the fetal heart rate could not be gated to trigger the MRI (poor arterial catheter pulsatility) or if the MRI was ceased earlier than expected due to animal welfare concerns as described previously (Cho *et al.*, 2024).

Re Line 301: Selecting a subset of 28 fetuses for Western blotting procedures was necessary, as this is the maximum number of samples that can fit onto a standard-size gel. We acknowledge that all samples could be run across more than one gel; however, this can introduce gel-to-gel variability. The selection of this subset was based on choosing fetuses that best matched the average phenotype of each group, as determined by weight and mean gestational fetal blood measures.

Line 405 for mean glucose concentrations can you specify days (pre and post LGUN or only after treatment started)
Additional text has been added to the results section to clarify that this is only after treatment started.

Line 410: During the treatment period, LGUN and LGUN+G had no impact on mean gestational maternal plasma glucose between treatment groups (Table 1).

Table 1 it is curious that the Hb and Hct don't correlate with one another better
Thank you for raising this. This observation is interesting as these two factors usually correlate proportionally but appear not to be in LGUN only. Upon reviewing this data, we have found a typographical error. The mean LGUN value for Hct was not **3**9.1; instead, the correct value was **2**9.1. This value has been updated in Table 1. Additional data within this table was also reviewed to ensure correctness.

References:

Al-Hasan YM, Evans LC, Pinkas GA, Dabkowski ER, Stanley WC & Thompson LP. (2013). Chronic hypoxia impairs cytochrome oxidase activity via oxidative stress in selected fetal Guinea pig organs. *Reprod Sci* **20,** 299-307.

Bloomfield FH, Oliver MH, Giannoulias CD, Gluckman PD, Harding JE & Challis JRG. (2003). Brief Undernutrition in Late-Gestation Sheep Programs the Hypothalamic-Pituitary-Adrenal Axis in Adult Offspring. *Endocrinology* **144,** 2933-2940.

Bloomfield FH, Oliver MH, Hawkins P, Holloway AC, Campbell M, Gluckman PD, Harding JE & Challis JR. (2004). Periconceptional undernutrition in sheep accelerates maturation of the fetal hypothalamic-pituitary-adrenal axis in late gestation. *Endocrinology* **145,** 4278-4285.

Camacho LE, Davis MA, Kelly AC, Steffens NR, Anderson MJ & Limesand SW. (2022). Prenatal Oxygen and Glucose Therapy Normalizes Insulin Secretion and Action in Growth-Restricted Fetal Sheep. *Endocrinology* **163**.

Cho SKS, Darby JRT, Saini BS, Holman SL, Lock MC, Perumal SR, Williams GK, Macgowan CK, Seed M & Morrison JL. (2024). Late-gestation maternal undernutrition induces circulatory redistribution while preserving uteroplacental function independent of fetal glycaemic state. *The Journal of Physiology* **n/a**.

Darby JRT, Chiu J, Regnault TRH & Morrison JL. (2022). Placental insufficiency induces a sexually dimorphic response in the expression of cardiac growth and metabolic signalling molecules upon exposure to a postnatal western diet in guinea pigs. *J Dev Orig Health Dis* **13,** 345-357.

Darby JRT, McMillen IC & Morrison JL. (2018). Maternal undernutrition in late gestation increases IGF2 signalling molecules and collagen deposition in the right ventricle of the fetal sheep heart. *J Physiol* **596,** 2345-2358.

Darby JRT, Zhang S, Holman SL, Muhlhausler BS, McMillen IC & Morrison JL. (2023). Cardiac growth and metabolism of the fetal sheep are not vulnerable to a 10 day increase in fetal glucose and insulin concentrations during late gestation. *Heliyon* **9,** e18292.

Dimasi CG, Darby JRT, Cho SKS, Saini BS, Holman SL, Meakin AS, Wiese MD, Macgowan CK, Seed M & Morrison JL. (2023). Reduced in utero substrate supply decreases mitochondrial abundance and alters the expression of metabolic signalling molecules in the fetal sheep heart. *The Journal of Physiology* **n/a**.

Dimasi CG, Darby JRT, Cho SKS, Saini BS, Holman SL, Meakin AS, Wiese MD, Macgowan CK, Seed M & Morrison JL. (2024). Reduced in utero substrate supply decreases

mitochondrial abundance and alters the expression of metabolic signalling molecules in the fetal sheep heart. *The Journal of Physiology* **602,** 5901-5922.

Grundy D. (2015). Principles and standards for reporting animal experiments in The Journal of Physiology and Experimental Physiology. *J Physiol* **593,** 2547-2549.

Lekatz LA, Caton JS, Taylor JB, Reynolds LP, Redmer DA & Vonnahme KA. (2010). Maternal selenium supplementation and timing of nutrient restriction in pregnant sheep: effects on maternal endocrine status and placental characteristics. *J Anim Sci* **88,** 955-971.

Long NM, Nijland MJ, Nathanielsz PW & Ford SP. (2010). The effect of early to mid-gestational nutrient restriction on female offspring fertility and hypothalamic-pituitary-adrenal axis response to stress. *J Anim Sci* **88,** 2029-2037.

McGillick EV, Morrison JL, McMillen IC & Orgeig S. (2014). Intrafetal glucose infusion alters glucocorticoid signaling and reduces surfactant protein mRNA expression in the lung of the late-gestation sheep fetus. *Am J Physiol Regul Integr Comp Physiol* **307,** R538-545.

Meakin AS, Darby JRT, Holman SL, Wiese MD & Morrison JL. (2022). Maternal-placental-fetal drug metabolism is altered by late gestation undernutrition in the pregnant ewe. *Life Sci* **298,** 120521.

Mühlhäusler BS, Adam CL, Marrocco EM, Findlay PA, Roberts CT, McFarlane JR, Kauter KG & McMillen IC. (2005). Impact of glucose infusion on the structural and functional characteristics of adipose tissue and on hypothalamic gene expression for appetite regulatory neuropeptides in the sheep fetus during late gestation. *J Physiol* **565,** 185-195.

Pereira SP, Tavares LC, Duarte AI, Baldeiras I, Cunha-Oliveira T, Martins JD, Santos MS, Maloyan A, Moreno AJ, Cox LA, Li C, Nathanielsz PW, Nijland MJ & Oliveira PJ. (2021). Sex-dependent vulnerability of fetal nonhuman primate cardiac mitochondria to moderate maternal nutrient reduction. *Clin Sci (Lond)* **135,** 1103-1126.

Regnault TR, Orbus RJ, Battaglia FC, Wilkening RB & Anthony RV. (1999). Altered arterial concentrations of placental hormones during maximal placental growth in a model of placental insufficiency. *J Endocrinol* **162,** 433-442.

Roman A, Desai N, Gupta M, Solanki M, Xue X, Chatterjee P, Rochelson B & Metz C. (2013). 403: Progesterone as a biomarker for intrauterine growth restriction in a rat model. *American Journal of Obstetrics and Gynecology* **208,** S177-S178.

Rozance PJ, Limesand SW, Barry JS, Brown LD & Hay WW, Jr. (2009). Glucose replacement to euglycemia causes hypoxia, acidosis, and decreased insulin secretion in fetal sheep with intrauterine growth restriction. *Pediatr Res* **65,** 72-78.

Smith KLM, Swiderska A, Lock MC, Graham L, Iswari W, Choudhary T, Thomas D, Kowash HM, Desforges M, Cottrell EC, Trafford AW, Giussani DA & Galli GLJ. (2022). Chronic developmental hypoxia alters mitochondrial oxidative capacity and reactive oxygen species production in the fetal rat heart in a sex-dependent manner. *J Pineal Res* **73,** e12821.

Song H, Polster BM & Thompson LP. (2021). Chronic hypoxia alters cardiac mitochondrial complex protein expression and activity in fetal guinea pigs in a sex-selective manner. *Am J Physiol Regul Integr Comp Physiol* **321,** R912-r924.

Steinhauser CB, Askelson K, Hobbs KC, Bazer FW & Satterfield MC. (2021). Maternal nutrient restriction alters thyroid hormone dynamics in placentae of sheep having small for gestational age fetuses. *Domest Anim Endocrinol* **77,** 106632.

Vonnahme KA, Hess BW, Hansen TR, McCormick RJ, Rule DC, Moss GE, Murdoch WJ, Nijland MJ, Skinner DC, Nathanielsz PW & Ford SP. (2003). Maternal undernutrition from early- to mid-gestation leads to growth retardation, cardiac ventricular hypertrophy, and increased liver weight in the fetal sheep. *Biol Reprod* **69,** 133-140.

Zhang H, Qi J, Wang Y, Sun J, Li Z, Sui L, Fan J, Liu C, Shang Y, Kong L & Kong Y. (2020). Progesterone Regulates Glucose Metabolism Through Glucose Transporter 1 to Promote Endometrial Receptivity. *Front Physiol* **11,** 543148.

Dear Janna,

Re: JP-RP-2025-288303R1 "Fetal glucose availability: A key regulator of the metabolic, hormonal and contractility profile of the fetal sheep heart" by Melanie Rose Bertossa, Jack RT Darby, Stacey L Holman, Steven KS Cho, Ashley S Meakin, Mitchell C Lock, Jordan Arthur Minns, Michael D Wiese, Christopher K Macgowan, Mike Seed, and Janna L Morrison

We are pleased to tell you that your paper has been accepted for publication in The Journal of Physiology.

The last Word (or similar) version of the manuscript provided will be used by the Production Editor to prepare your proof. When this is ready you will receive an email containing a link to Wiley's Online Proofing System. The proof should be thoroughly checked and corrected as promptly as possible.

Authors should note that it is too late at this point to offer corrections prior to proofreading. Major corrections at proof stage, such as changes to figures, will be referred to the Editors for approval before they can be incorporated. Only minor changes, such as to style and consistency, should be made at proof stage. Changes that need to be made after proof stage will usually require a formal correction notice.

All queries at proof stage should be sent to: TJP@wiley.com.

Following copyediting and typesetting the accepted version will be published online.

Best wishes,

Bjorn Knollmann
Senior Editor
The Journal of Physiology

IMPORTANT POINTS TO NOTE FOLLOWING YOUR PAPER'S ACCEPTANCE:

If you would like to receive our 'Research Roundup', a monthly newsletter highlighting the cutting-edge research published in The Physiological Society's family of journals (The Journal of Physiology, Experimental Physiology, Physiological Reports, The Journal of Nutritional Physiology and The Journal of Precision Medicine: Health and Disease), please click this link, fill in your name and email address and select 'Research Roundup':
https://www.physoc.org/journals-and-media/membernews

• TRANSPARENT PEER REVIEW POLICY: To improve the transparency of its peer review process, The Journal of Physiology publishes online as supporting information the peer review history of all articles accepted for publication. Readers will have access to decision letters, including Editors' comments and referee reports, for each version of the manuscript as well as any author responses to peer review comments. Referees can decide whether or not they wish to be named on the peer review history document.

• You can help your research get the attention it deserves! Check out Wiley's free Promotion Guide for best-practice recommendations for promoting your work at: www.wileyauthors.com/eeo/guide. You can learn more about Wiley Editing Services which offers professional video, design, and writing services to create shareable video abstracts, infographics, conference posters, lay summaries, and research news stories for your research at: www.wileyauthors.com/eeo/promotion.

• IMPORTANT NOTICE ABOUT OPEN ACCESS: To assist authors whose funding agencies mandate public access to published research findings sooner than 12 months after publication, The Journal of Physiology allows authors to pay an Open Access (OA) fee to have their papers made freely available immediately on publication.

The Corresponding Author will receive an email from Wiley with details on how to register or log-in to Wiley Authors Services where you will be able to place an order.

• You can check if your funder or institution has a Wiley Open Access Account here: https://authorservices.wiley.com/author-resources/Journal-Authors/licensing-and-open-access/open-access/author-compliance-tool.html.

EDITOR COMMENTS

Reviewing Editor:

Thank you for addressing the comments/concerns raised. Congratulations on an important contribution to the field.

Senior Editor:

The work is now acceptable. Excellent contribution to the Journal!

REFEREE COMMENTS

Referee #1:

Thank you for addressing my comments. The paper is much improved.

Referee #2:

The points have been addressed sufficiently.
